# Investigating the Consequences of Interference between Multiple CD8^+^ T Cell Escape Mutations in Early HIV Infection

**DOI:** 10.1371/journal.pcbi.1004721

**Published:** 2016-02-01

**Authors:** Victor Garcia, Marcus W. Feldman, Roland R. Regoes

**Affiliations:** 1 Institute of Integrative Biology, ETH Zurich, Zurich, Switzerland; 2 Department of Biological Sciences, Stanford University, Stanford, California, United States of America; Los Alamos National Laboratory, UNITED STATES

## Abstract

During early human immunodeficiency virus (HIV) infection multiple CD8^+^ T cell responses are elicited almost simultaneously. These responses exert strong selective pressures on different parts of HIV’s genome, and select for mutations that escape recognition and are thus beneficial to the virus. Some studies reveal that the later these escape mutations emerge, the more slowly they go to fixation. This pattern of *escape rate decrease*(ERD) can arise by distinct mechanisms. In particular, in large populations with high beneficial mutation rates interference among different escape strains –an effect that can emerge in evolution with asexual reproduction and results in delayed fixation times of beneficial mutations compared to sexual reproduction– could significantly impact the escape rates of mutations. In this paper, we investigated how interference between these concurrent escape mutations affects their escape rates in systems with multiple epitopes, and whether it could be a source of the ERD pattern. To address these issues, we developed a multilocus Wright-Fisher model of HIV dynamics with selection, mutation and recombination, serving as a null-model for interference. We also derived an interference-free null model assuming initial neutral evolution before immune response elicitation. We found that interference between several equally selectively advantageous mutations can generate the observed ERD pattern. We also found that the number of loci, as well as recombination rates substantially affect ERD. These effects can be explained by the underexponential decline of escape rates over time. Lastly, we found that the observed ERD pattern in HIV infected individuals is consistent with both independent, interference-free mutations as well as interference effects. Our results confirm that interference effects should be considered when analyzing HIV escape mutations. The challenge in estimating escape rates and mutation-associated selective coefficients posed by interference effects cannot simply be overcome by improved sampling frequencies or sizes. This problem is a consequence of the fundamental shortcomings of current estimation techniques under interference regimes. Hence, accounting for the stochastic nature of competition between mutations demands novel estimation methodologies based on the analysis of HIV strains, rather than mutation frequencies.

## Author Summary

Within-host evolution of human immunodeficiency virus (HIV) is shaped by strong immune responses mounted against the virus. Multiple CD8^+^ T cell populations, each recognizing a specific part of an HIV protein, simultaneously suppress HIV growth. Escape mutations that arise in HIV genome regions coding for these virion protein parts, impair CD8^+^ T cell recognition and are consequently strongly selected. The emergence and rise of these escape mutations exhibits an intriguing temporal pattern: the earlier an escape mutation arises, the faster it goes to fixation. This pattern is termed escape rate decrease (ERD). In this paper, we tested computationally whether interference, i.e. the coexistence of multiple genetically distinct HIV strains engaged in competitive interaction within the host, could be a possible source of ERD. As an alternative, we also mathematically derived the temporal pattern of escapes under interference-free conditions, and compared this with data. We found that interference between multiple beneficial mutations could generate ERD. However, ERD does not imply the presence of interference. Thus, more detailed data is required to unambiguously determine whether interference effects influence ERD generation. Nevertheless, interference should be considered when studying the within-host evolution of HIV. Ignoring its effects on population dynamics can severely underestimate the protective capacity of CD8^+^ T cells.

## Introduction

Strong immune responses both drive and shape the early within-host evolution of acute Human Immunodeficiency virus (HIV) during infection [1]. The earliest of these responses are mediated by CD8^+^ T cells, which exert selective pressures on the virus that are important to the clinical outcome of infections [2]. A precise quantification of their protective capacity for the host is of great relevance, and could inform HIV vaccine design [3–5].

The significance of CD8^+^ T cells for the control of viral replication is supported by ample experimental evidence [4, 6–14]. One of the most important effector functions of CD8^+^ T cells is to recognize and kill viral target cells (CD4^+^ T cells) that present viral peptides, termed *epitopes*, on their surface, thereby signaling the presence of intra-cellular pathogens [15–17]. Mutant viral strains that code for epitopes that are sufficiently distinct from those recognized by CD8^+^ T cells, avoid clearance and so receive a substantial selective advantage [3, 4, 18]. This phenomenon is termed *escape*. Mutations that escape from the effects of CD8^+^ T cells are a recurring phenomenon in HIV and can, in some individuals, be observed multiple times during acute infection [19, 20].

Escape mutations occur at multiple sites in the viral genome [19–21]. The presence of several distinct escape mutations during early infection (which we define to last up to ∼4 months) implies that CD8^+^ T cell pressures act on multiple epitopes simultaneously [20, 22].

The selective pressure exterted via the epitope-specific CD8^+^ T cells influences how fast escape mutations go to fixation. These *escape rates* are commonly estimated under the assumption of independence between escape mutations: Irrespective of the presence of other escape mutations, the escape rate of an individual escape mutation is assessed by fitting a logistic curve to its frequency time course [22–26]. The CD8^+^ T cell killing efficacy is then approximated by the estimated escape rate plus an offset arising from fitness costs [24]. Fitting such curves to sample points of the fixation trajectories of mutations in different epitopes shows a pattern of *escape rate decrease*: late-emerging escape mutations have lower escape rates (that is, they go to fixation more slowly) than early-emerging escape mutations [22, 24, 26].

Escape rate decrease (ERD) could be generated by different independent mechanisms. In particular, it has been hypothesized to partly stem from effects resulting from HIV’s large population size and high mutation rate [27–31]. In this scenario, beneficial mutations, such as escape mutations, are likely to arise simultaneously at distinct sites in HIV’s genome [32, 33]. Since selection dominates the early HIV evolution within patients, beneficial mutations should thus start to rise in frequency [33]. Eventually, they will enter a state of competition, delaying each other’s fixation [34]. Such delays are generated by the mutually inflicted growth impairment among beneficial mutations, termed *interference* [35–37]. As a consequence, the fixation trajectories of individual escape mutations could become arranged in such way that they give rise to ERD [27].

Whether interference is prevalent enough during early HIV infection to cause or amplify phenomena like ERD remains an open question. On the one hand, given the low recombination rates [38, 39] in relation to the selective pressures in early HIV infection, theoretical population genetics suggests that abundant beneficial mutations will generate multiple concurrent lineages [1, 33, 35–37, 40–42]. Furthermore, the presence of interference effects in HIV is corroborated by some recent studies. In one instance, haplotype reconstruction techniques have revealed evidence for interference in one patient during early HIV infection [43]. The recently observed phenomenon of *epitope shattering*, is a further strong indication for interference at the intra-epitope level [2, 22, 44, 45].

On the other hand, some studies suggest that HIV evolves by the sequential fixation of beneficial mutations [46], which is commonly associated with a lack of interference [33]. This is assumed to be the result of a severe bottleneck in population size during transmission in combination with strong selection [47].

Despite the uncertainty about its role in early HIV evolution, neglecting interference effects could presuppose an undervaluation of the protective role of CD8^+^ T cells. In interference regimes, the application of logistic curve fitting to escape data will produce biased estimates of killing efficacies, since beneficial mutations do not behave independently. In previous work [27], we have shown that this scenario can unfold in a realistic model of HIV dynamics that incorporates escapes at two loci, leading to severe underestimates of CD8^+^ T cell killing efficacies.

In this study, we investigated whether features of the ERD pattern found in data are consistent with a simple model of interference [33] or alternatively, an interference-free model. To model interference, we developed a discrete-generation Wright-Fisher model with selection and multiple loci. Controlling population sizes, we then studied how increasing levels of interference affect ERD in simulations of early HIV infection under a variety of conditions. Selective advantages were assumed to be equal for all beneficial mutations. Besides different population sizes, simulations were run for different time spans of neutral evolution preceding immune-mediated selection, as well as different rates of recombination and numbers of loci.

To assess the effects of alternative mechanisms that preclude interference, we derived predictions for the pattern of ERD under the assumption of free recombination and the presence of standing variation. We then assessed whether both models could replicate the ERD pattern features from data obtained in [26].

We found that interference consistently produces ERD patterns of similar magnitude and shape to those found in [26] in parameter regimes commonly associated with early HIV infection. How the number of loci, recombination rates and neutral evolution phases affect ERD in the multiple mutations regime is well explained by the underexponential decline of escape rates over time. Surprisingly, both the models with and without interference were able to replicate the underexponential decline pattern of patient ERD.

## Materials and Methods

To investigate the effects of interference on the escape rates of beneficial mutations, we implemented a Wright-Fisher model with multiple loci, recombination and time-delayed selection. Our model is similar in spirit to FFPopSim [48] employed in the exploration of early HIV infection dynamics [34, 49].

The model simulates the number of target cells infected with specific viral strains represented as haplotypes **i** = *i*_1_ *i*_2_ … *i*_*L*_ with *L* loci, where *i*_*k*_ ∈ {0, 1}. Infection is initiated with a single ancestral or wildtype haplotype, as in the majority of infections [50–52], where by default: *i*_*k*_ = 0 ∀*k*. The abundance *n*(**i**) of each haplotype **i** is tracked at each generation, where *N* = ∑_**i**_ *n*(**i**) is the population size. If haplotype **i** has 1 at a particular position *j*, it is assumed to carry a mutation that confers an additive selective fitness advantage *s*. No epistatic effects among beneficial mutations are considered, and we neglect deleterious mutations.

Haplotypes can mutate into other haplotypes. We assume a mutation rate per locus per generation of *μ*_*b*_ = 10^−4^ [34]. Only forward mutations, i. e. conversion from zeroes to ones in the binary sequences, are considered.

The reproductive scheme is divided into two stages. The first stage is an expansion phase, where we assume the absence of selection, termed *neutral phase* in the following. In the neutral phase, one infected cell is assumed to infect a Poisson distributed number of new cells. The mean of the distribution is *R*_0_ = 8, the best estimate for the basic reproductive number of HIV [53, 54]. The initial population of cells *N*(*t* = 0) is one. The generation time of the virus for replication in a cell is set to two days [30, 55–57]. When the total number of infected cells reaches a predefined upper limit *N*, the population is resampled without selection at the constant population size *N*. We modeled neutral phase duration lengths of *τ*_*n*_ ∈ {0, 20, 28} days. Although *τ*_*n*_ = 0 is an unrealistic assumption, it can reveal whether the standing variation that accumulates when *τ*_*n*_ > 0, is the sole source of ERD.

In the modeling of the neutral phase we follow [50, 52], which ignore the consequences of the collapse of viral load during the acute phase on the grounds that this level of biological realism suffices to study how the diversity of the population changes. A similar approach is employed in other studies [48, 49].

In the second phase, the simulation proceeds by resampling the existing population of size *N* with selection. We explored values *N* ∈ {10^2^,5 × 10^2^, 10^3^, …, 10^5^}. These values range over estimates of the effective population size *N*_*e*_ of HIV in its acute and chronic phases [46, 50, 52, 58–61].

Selection acts by modifying the resampling probability of haplotypes depending on their pre-assigned fitness and the average fitness of the population. Strain **i** is assigned a selective advantage, *s*_**i**_, which represents the exponential growth rate of that strain [33]. The growth factor per generation or *fitness* is given by $w_{i}=e^{s_{i}}$. For example, a strain with three escape mutations (each with fitness advantage *s*) has a fitness of *w* = *e* ^3*s*^ relative to the haplotype with all zeroes. Hence, strain **i** is sampled with probability $p_{i,s}=\frac{e^{s_{i}}}{\langle e^{s}\rangle }\cdot p_{i}$, where $\langle e^{s}\rangle =\sum_{i}e^{s_{i}}p_{i}$ is the average fitness, and *p*_**i**_ is the fraction of strains of type **i** in the population [48, 62]

Recombination occurs only in cells infected by virions stemming from multiply infected cells. The fraction of multiply infected cells is held constant at 1% [38, 39, 63–65]. The process of recombination is implemented with as much biological detail as possible, including formation of pairs of different strains and the action of reverse transcriptase (see S1 Text, S1 and S2 Figs). The template switching rate was assumed to be *ρ* ≈ 3 × 10^−4^ per base pair per generation [66, 67].

In order to implement the model, we used the C# programming language. Simulations were run up to 2000 generations, corresponding to 4000 days of infection.

### Escape dynamics without interference

A beneficial mutation’s frequency time course is assumed to follow a logistic curve when going to fixation in the absence of interference [68]. This population genetic result has been rederived and used in the context of HIV dynamics [22–24, 26]. In the notation introduced by [26], the frequency time course *f*(*t*) of a beneficial mutation is:

$$f\left(t\right)=\frac{f_{0}}{f_{0}+\left(1-f_{0}\right)e^{-\epsilon t}},$$ (1)

where *f*_0_ is the initial frequency of the mutant and *ϵ* is the average mutant fitness advantage. *ϵ* is termed the *escape rate* of the mutant, which is commonly used as a measure for the growth advantage that an escape mutation confers to its carrier.

Besides the escape rate, another useful and intuitively accessible parameter is the time for the mutant frequency to reach fifty percent:

$$\tau_{50}=\frac{1}{\epsilon }\ln\left(\frac{1-f_{0}}{f_{0}}\right).$$ (2)

This parameter is termed the *escape time*. Given *ϵ* and *τ*_50_, the logistic escape curve Eq (1) is fully determined. Both *ϵ* and *τ*_50_ must be inferred from experimental data.

### Calculation of escape rate decrease

In simulations, we calculated the ERD by following methods that are commonly employed in experimental studies. Within each simulation run, we determined the trajectory of each mutated epitope’s frequency over time. Mimicking experimental procedures, we sampled these frequencies at predetermined time points similar to those chosen in Goonetilleke et al. [20]—densely sampling in the beginning, and sampling more sparsely late in infection. If not stated otherwise, the sampling times were 0, 10, 20, 50, 100, 190 and 300 days after the onset of selection. To further approximate empirical settings, simulations were capped at 400 days.

In each simulation, we then individually fitted Eq (1) to every epitope-mutation that had gone to fixation, as in [22, 24, 26]. Each fit for a beneficial mutation going to fixation provides a pair of estimates for the escape rate *ϵ* and the escape time *τ*_50_. Repeated fits lead to a set of pairs (*τ*_50,*l*_, *ϵ*_*l*_) for each locus or epitope *l* ∈ {1, …, *L*}.

These values were then used to calculate an association between successive escape rates and escape times (see S3 Fig for two simulation examples involving escapes, logistic model fits, and the escape rate and time association). This was achieved by fitting a linear regression formula log_10_(*ϵ*) = *a* + *b* ⋅ *τ*_50_ to the data pairs (*τ*_50,*l*_, *ϵ*_*l*_). This is mathematically equivalent to the regression log_10_(*ϵ*/*a*′) = *b* ⋅ *τ*_50_, with *a* = log_10_(*a*′) and *a*′ > 0. The slope of this regression is the value of the parameter *b*, termed the value of *escape rate decrease* (ERD value). The more negative this value, the more pronounced the ERD. If the ERD value is zero, there is no escape rate decrease between subsequent escape rates. The logarithm of a value having units, such as log_10_(*ϵ*/*a*′), does not have a well-defined unit itself, and will thus be treated as unitless. Therefore escape rate decrease values *b* will be used with units of *day* ^−1^.

## Results

### Interference is likely in HIV in tight linkage regimes

When beneficial mutations are rare in a population of asexually reproducing individuals, they commonly will not overlap temporally, and will go to fixation in a sequential manner. However, if they are common, different beneficial mutations will be present simultaneously in the population, and the periods in which they grow in frequency will likely coincide. Assuming that selection dominates drift, the fixation trajectories of the mutant frequencies will thus be affected by the presence of other, concurrent mutations, i. e. they will *interfere*. The fixation time—the time at which a beneficial mutation is present in all individuals of a population– of one single mutation will be delayed with respect to the concurrence-free scenario.

Mathematically, a beneficial mutation is defined to interfere with another whenever its establishment time –the average time for a mutation to become so prevalent that it is assumed to go to fixation deterministically– is smaller than the average fixation time [33]. In population genetics, this is called the “concurrent mutations regime” [33]. Desai and Fisher distinguish two aspects of the concurrent mutations regime: clonal interference [32] as well as multiple mutations [33] effects. Here, we will focus on the multiple mutations case only, where each mutation is assumed to confer the same selective advantage *s*. Due to its simplicity, this case serves as a useful null-model for interference. If a system is in the multiple mutations regime, it will also display interference.

Whether interference occurs in the adaptive evolution of an asexual system is assessed by the measurement of three parameters: the population size *N*, the beneficial mutation rate per generation per individual (or sequence) *μ*_*b*_ and the fitness advantage *s* associated with such a beneficial mutation. The system is in the concurrent mutations regime if [32, 33]:

$$N\mu_{b}≳\frac{1}{\ln(Ns)}.$$ (3)

From this inequality it follows that increasing the population size *N* makes interference more probable if *μ*_*b*_ and *s* are held fixed. In fact, we can interpret Eq (3) as defining a threshold value for *N*, *N*_0_(*μ*_*b*_, *s*), above which interference should be the norm in a system, and below which *sequential fixations* should be prevalent.

**Fig 1:**
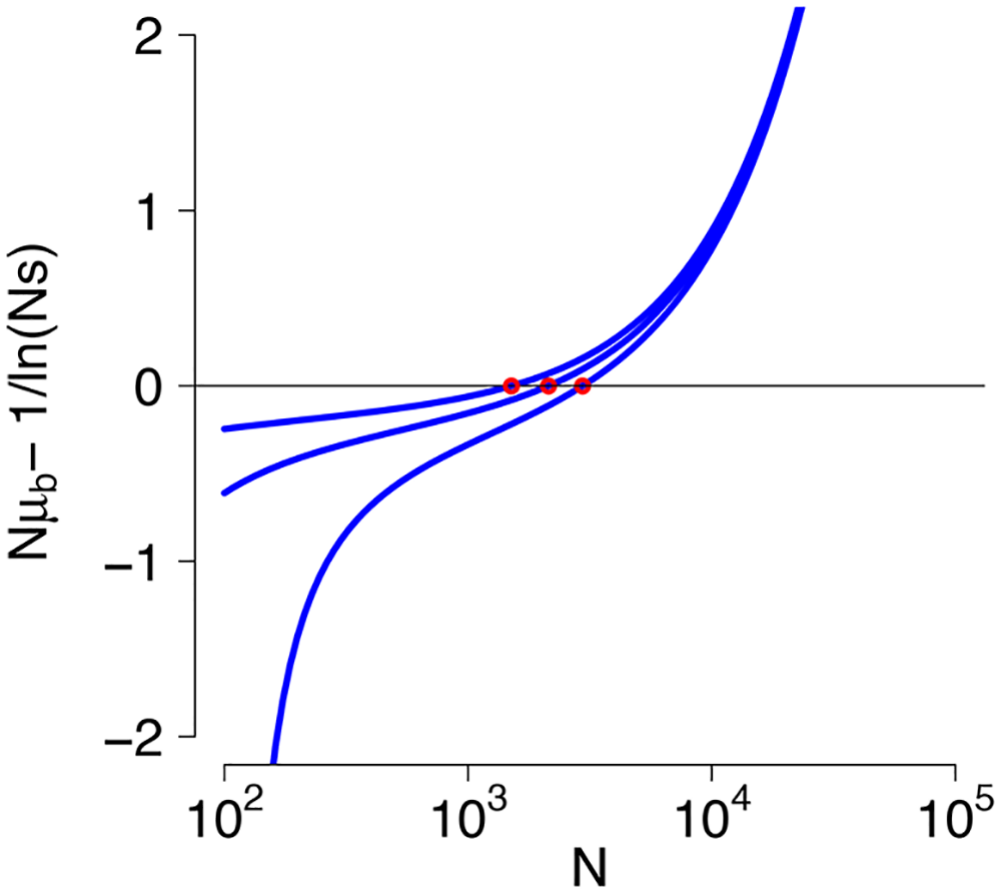
HIV within-host evolution is likely to be affected by interference effects. Examples of the function $N\mu_{b}-\frac{1}{\ln(Ns)}$ over a wide range of values of *N* for *μ*_*b*_ = 10^−4^ and for *s* = {0.01, 0.05, 0.5}. The red points are the threshold values *N*_0_ for each example function. *N*_0_ varies only by about one order of magnitude over the range of relevant HIV selection coefficients.

Fig 1 shows the difference between *Nμ*_*b*_ and $\frac{1}{\ln(Ns)}$ over a wide range of values of *N* and for different values of *s*. The curves have a zero at *N*_0_, which defines a threshold for the transition from the sequential fixations to the concurrent mutations regime at higher *N* [33]. For the parameters used to model interference in this study, *μ*_*b*_ = 10^−4^ and *s* = 0.5, we obtain *N*_0_ ≈ 1.5 ⋅ 10^3^.

Although this theory can only be applied indirectly to HIV –since HIV exhibits some recombination it breaks the prerequisite of asexual evolution– the above considerations can lead to useful insights. Assuming that due to strong selective pressures HIV is quasi-asexual, most estimates for *N* and *s* found in the literature should place HIV in the concurrent mutations regime during early infection, right of *N*_0_ part of Fig 1. The estimates for HIV’s effective population size lie between 10^4^ [52, 60, 61] and 10^7^ [28, 29, 69] during the asymptomatic phase. The values used in models that focus on early HIV infection tend to have a broader range, namely between 10^2^ and 10^5^ [46, 52, 70]. The selection coefficients range from *s* = 0.81 [24, 46] to *s* = 0.01 [20, 24, 26]. Thus, these considerations raise the question whether interference effects are consistent with common estimates found the HIV literature, and ERD in particular.

### Escape rate decrease pattern is amplified and stabilized by increasing interference

To investigate whether interference could cause ERD in escapes occurring simultaneously at multiple loci, we adopted a simple strategy: In simulation experiments, we tracked how ERD changed with increasing population size. According to the population genetic theory outlined above, a system must transition from a sequential fixations to a multiple mutations regime, where interference effects are present. If interference causes ERD, we would thus expect pronounced ERD to emerge as the system enters an interference regime.

To track ERD, we utilized a Wright-Fisher model of early HIV infection that incorporates multiple epitopes or loci, selection, mutation and recombination (see Materials and Methods). We examined the regime transition under different recombination rates, different neutral phase lengths and different numbers of loci. Distinct effective recombination rates were achieved by varying the genomic distances between loci coding for beneficial mutations on the genome. The number of loci considered is in accordance with the empirically observed number of CD8^+^ T cell clones simultaneously targeting distinct epitopes [20, 22, 71–73] (see Materials and Methods). For each combination of parameter values considered, 100 simulation repeats were run. To be able to compare the ERD values in the simulations with data, we adopted the empirical sampling scheme described in *Materials and Methods*.

**Fig 2:**
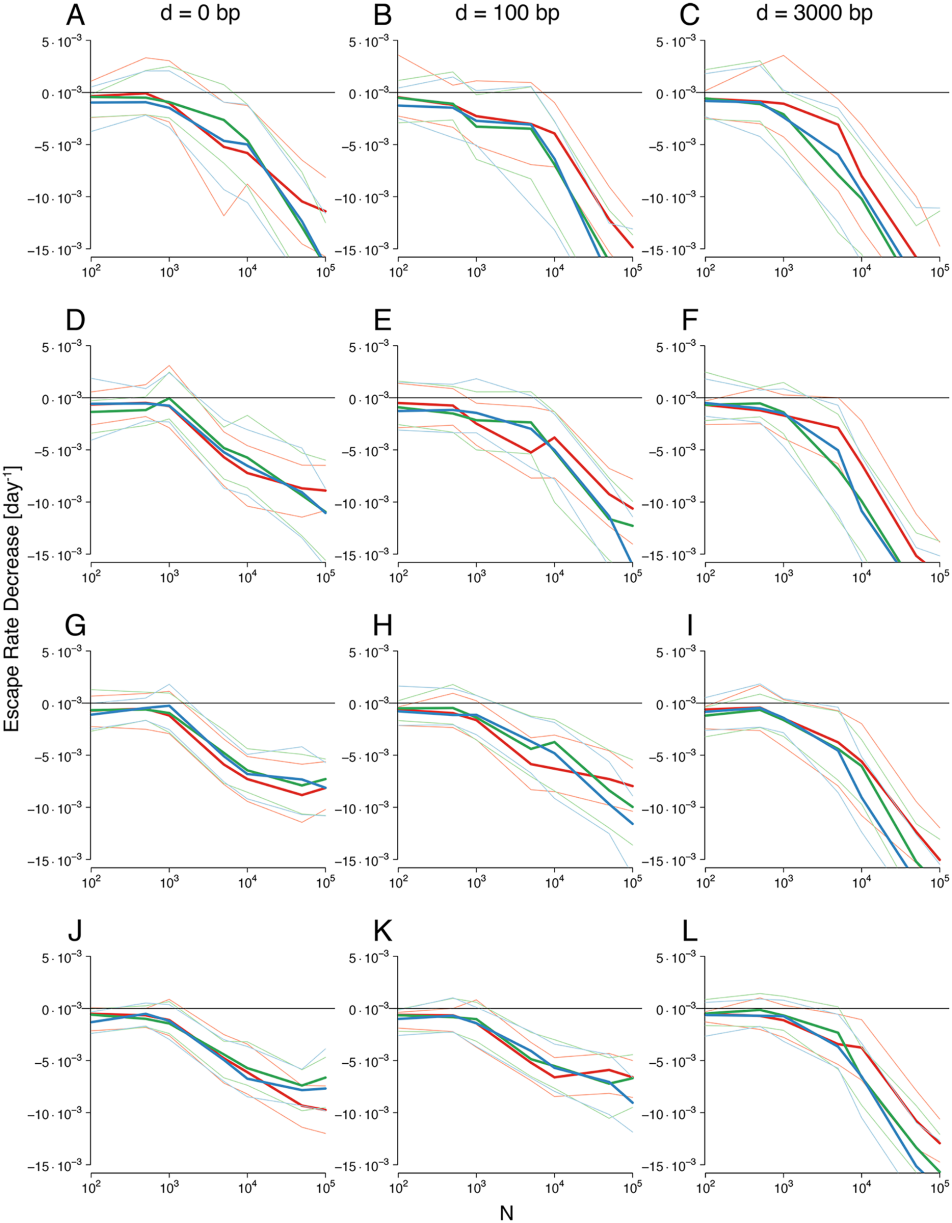
ERD values versus population sizes and different neutral phase lengths, linkage strengths and number of loci. Rows: Simulations were performed for *L* = 3, 4, 5, 6 loci shown in rows A-C, D-F, G-I and J-L, respectively. Columns: The effect of loosening linkage on ERD is shown for inter-mutation distances of *d* = 0 (complete linkage), *d* = 100 and *d* = 3000 nucleotides (nt) or base pairs (bp). The red line (green, blue lines) and the lines of light-red (light-green, light-blue) color show the median and 25 and 75 percentile ranges of ERD inferred from simulations with neutral phase of 0 (20, 28) days, respectively. Selection coefficients are *s* = 0.5 for all beneficial mutations, and the epitope mutation rate is *μ*_*b*_ = 10^−4^ per locus per generation. Samples were taken as described in *Materials and Methods*, mimicking empirical sampling schemes.

We observed that in all examined scenarios, as the system transitions into the interference regime, the ERD values become negative, indicating more pronounced ERD (see Fig 2). This corroborates the notion that interference does cause ERD in our null-model system. This effect persists across realistic parameter ranges. In particular, over the range of populations sizes examined, the higher the population size *N*, the more interference we expected, and the more negative the ERD values measured in simulations were.

These results have to be interpreted in the context of empirical ERD values. The ERD values in the patients analyzed in [20], based on the data presented in the Supplementary Material of [26] are comparable in magnitude, as reported in [27]: For escapes with *τ*_50_ within the first two years, values are about −0.006 day^−1^ (CH44), −0.008 day^−1^ (CH77) and −0.01 day^−1^ (CH58). These values are only reached at population sizes of about *N* = 10^4^−10^5^ in our simulations.

We also tested whether the goodness of the logistic model fits was affected by the emergence of interference. As a goodness of fit statistic, we used the medians across simulation repeats of the medians of the residual sum of squares (RSS) of logistic fits across epitopes. The goodness of fit worsened as more interference was attained (see S4 Fig). As expected, recombination markedly improved fit quality. Higher levels of standing variation should lead to more interference (see below). Thus, shorter phases of neutral expansion also improved fit quality, which we hypothesize is explained by the reduction of standing variation.

In the following, we examine and explain how sampling schemes, the number of loci, recombination rates and neutral phase lengths affect ERD values. We further analyze whether ERD values can be assumed to significantly differ from zero.

#### Interference also causes ERD using equally-spaced sampling

To test whether the appearance of negative ERD values in interference regimes is not an artifact of the experimental sampling times, we compared ERD values inferred by mimicking experimental procedures to values obtained by different procedures. We found that in general, the behavior of ERD values under fixed-period sampling schemes is very similar to that shown in Fig 2 (see S1 Text, S5 Fig).

To examine this phenomenon more closely, we explored whether ERD values obtained by different sampling schemes tend to correlate (see S6 Fig). When comparing the ERD values determined by the empirical sampling scheme, *b*_*e*_, with ERD values found by sampling roughly every 13 days, *b*_*c*_, we found that the correlation coefficient could range from 0.26 to 0.7. We then applied two types of regression to analyze how *b*_*c*_ depends on *b*_*e*_, a standard linear regression, and a Theil-Sen regression. The null hypothesis that the slope of the regression line is equal to one is rejected in one third of the cases, for both linear and Theil-Sen regressions (confidence intervals do not contain value one). The null hypothesis that the slope is zero is always rejected.

These results suggest that the appearance of ERD when entering the multiple mutations regime cannot be attributed to experimental sampling times alone.

#### More loci and higher recombination amplify ERD by changing escape times

Fig 2 shows that, with increasing numbers of loci, ERD values increase, that is, ERD becomes less pronounced. It also shows that more recombination leads to more negative ERD values, or more pronounced ERD. These results are counter-intuitive, since interference effects are expected to be amplified with more loci [42] and dampened with more recombination.

This behavior arises from the nonlinear change in the logarithm of escape rates (*log-escape rate*) over escape time, which will be discussed in more detail below. The inferred escape rates do not decrease in an exponential fashion over escape time, but decay more slowly, in an underexponential way. They thus show a very marked decline early after the onset of selection, and a slower decline later on.

**Fig 3:**
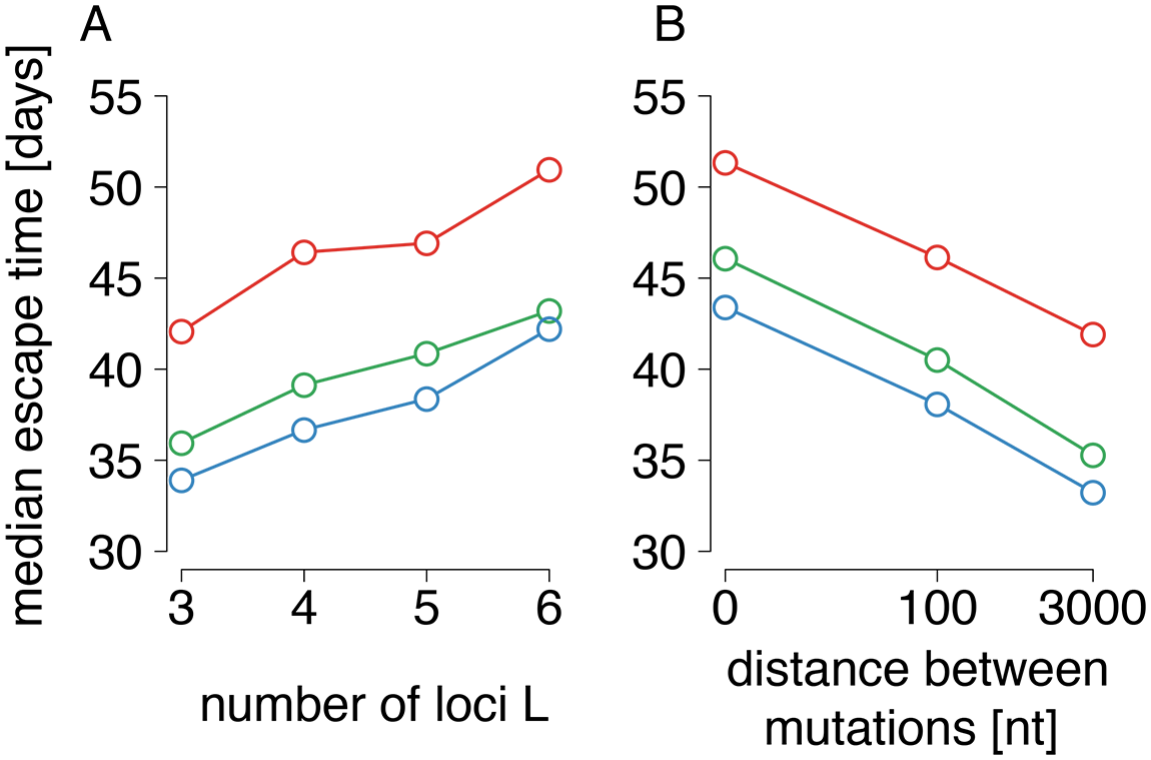
Median escape times estimated from simulations for different numbers of loci, neutral phase lengths and recombination rates. A: Effect on median escape times of varying numbers of loci *L* in simulations. Here, the distance between mutations is fixed at *d* = 100 nt. B: Effect of loosening linkage for inter-mutation distances of *d* = 0 (complete linkage), *d* = 100 and *d* = 3000 nt, for a fixed number of loci *L* = 5. Colors: The red, green, and blue lines denote the median escape times inferred from simulations with neutral phase of 0, 20, and 28 days, respectively. The median escape times were inferred from the aggregate values of a previous averaging process: For each simulation, the median of the escape times is found (see Materials and Methods) for experiment-like sample times. These values were gathered from 100 simulation repeats, for which a second median is calculated, displayed in the figure. Selection coefficients and mutation rates as in Fig 2. The patterns in A) and B) are retained for different values of fixed *d* and *L*, see S7 Fig.

Due to the non-linearity of the escape time and log-escape rate association, the times at which the escapes emerge has an important effect on the inferred ERD value. To study this effect, we analyzed the escape times from Fig 2 in more detail. Fig 3 shows how the median of the escape times across simulations (inferred from sampling times roughly as in [20]), is affected by the number of loci, the neutral phase length and the recombination rate.

**Fig 4:**
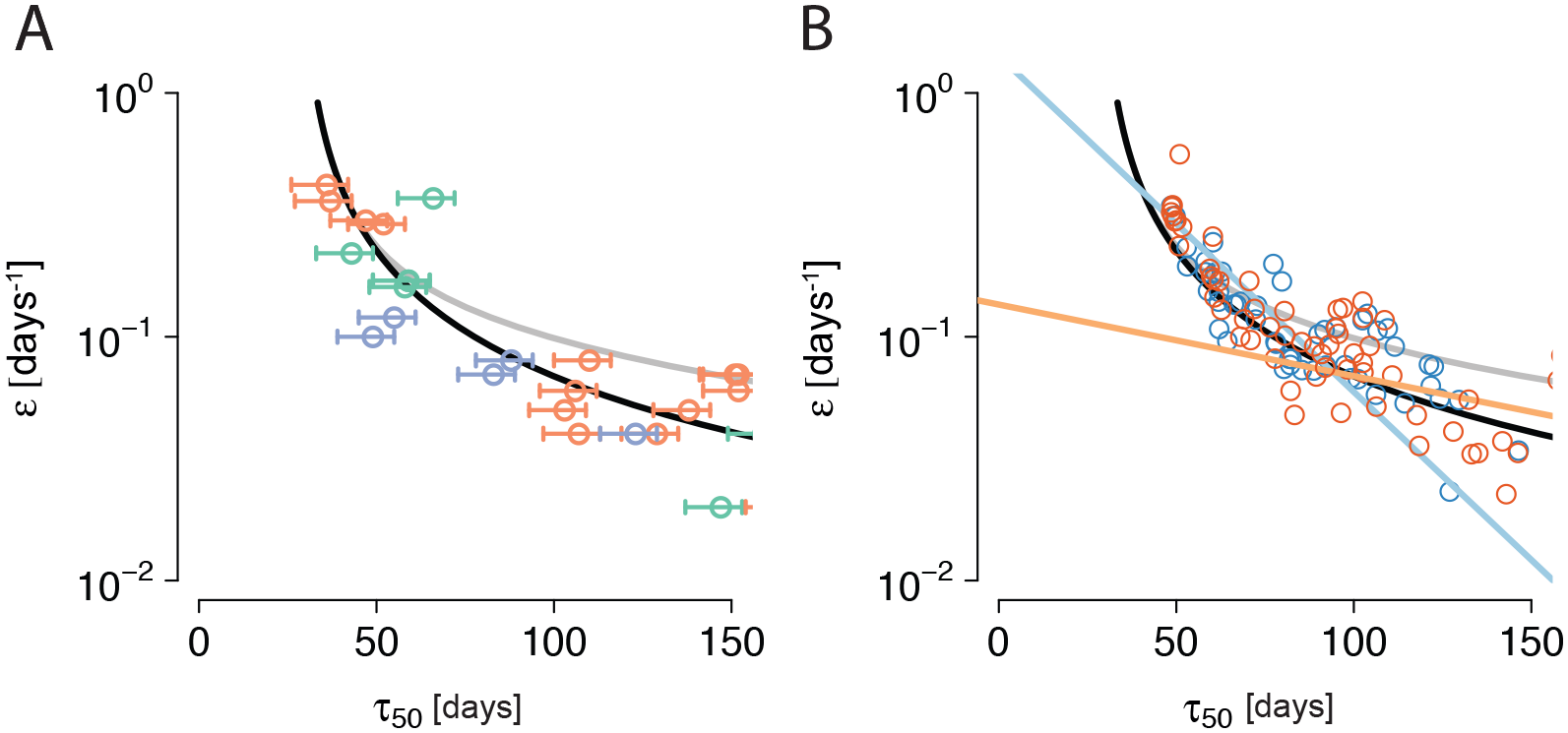
Comparison of inferred escape rates for two systems with A) free and B) no recombination. A) The data points are escape rates inferred by Ganusov et al (see [26], Supplementary Material) for patients CH40 (orange points), CH58 (violet points) and CH77 (green points). All the patients were identified to be in Fiebig stage II [20], lasting between day 18 and 34 post infection (on average, 22 days post infection) [52]. This relative uncertainty is captured by the error bars of the escape times. The curves are the expectation in the absence of interference, given by Eq (10), where *N* = 10^4^ (black) and *N* = 10^3^ (grey), and *τ*_*n*_ = 28 days. B) Blue and red points: Inferred escape rates from twenty simulations (10 and 10, respectively) of an asexual population of HIV strains with five and six loci, respectively, each locus conferring an additive selective advantage of *s* = 0.5. The neutral phase in the population was set to 28 days, the population size simulated was *N* = 10^4^ and full linkage was assumed (*d* = 0). For the inference of escape rates, similar sampling times as in [20] were used (which are also the sampling times used for A). The curves are identical to those shown in panel A. The light blue and orange lines are two example ERD regressions log_10_(*ϵ*) = *a* + *b* ⋅ *τ*_50_ through the escape rates of two simulations with five and six loci, respectively.

Fig 3 offers an explanation for both counter-intuitive effects found in simulations. On the one hand, Fig 3A) shows that escapes from simulations with fewer loci are more likely to emerge at earlier times, where the decline in escape rates is steeper (see Fig 4B, blue points). This leads the regression line through these points to have a very negative slope, which is equivalent to strongly negative ERD values (see Fig 4B, light blue line). More loci cause escape times to be more spread out due to interference, placing some escape times in regions where the escape rates decline more slowly, thus leading to larger ERD values (see Fig 4B, red points and orange line). The distributions of escape times across all considered cases is shown in S7 Fig.

On the other hand, Fig 3 also explains why we also found that increasing recombination rates lead to more negative ERD values. As is visible in Fig 3B), more recombination (larger distance between mutations) accelerates the emergence of escapes, reducing interference. Thus, the escape times become more closely clustered in the phase where escape rates decline rapidly, and ERD values become more negative.

#### Small effects of duration of neutral phase preceding selection on ERD

In general, we would expect simulations with more extended neutral periods to produce more ERD, since the potential for interference is increased by standing variation at the onset of selection [74]. However, the effect of the neutral periods is small compared to the impact of numbers of loci and recombination. Except for the case of an inter-locus distance of *d* = 3000 nt, where this expectation is verified, we were not able to observe a clear trend in the direction of the expectation.

Simulations with a neutral phase of 0 days do not exhibit standing variation before the onset of selection. Thus, we conclude that in our simulations the emergence of ERD cannot be attributed to standing variation alone, but must be associated with the transition to an interference regime.

#### Decreasing ERD-regression p-values for increasing interference

In order to investigate the reliability of the ERD estimates, we also tested whether the ERD values significantly differed from zero in all of the cases shown in Fig 2. More specifically, we investigated whether the association between log-escapes rates and escape times—which underlies the measure of escape rate decrease– was statistically significant (at a significance level of 5%) in the simulations.

To this end, we tested one particular null hypothesis in every linear regression performed on the escapes occurring within a single simulation run. The null hypothesis states that *b* = 0 in the regression formula *log*_10_(*ϵ*/*a*′) = *b* ⋅ *τ*_50_ used for ERD calculation, and thus, that the ERD value is zero.

For each simulation, we calculated the p-value for this null hypothesis (see S3 Fig for examples). As expected, ERD values for low population sizes were close to zero. At low population sizes, beneficial mutations go to fixation predominantly sequentially and, because their fitness advantage is identical, ERD is not pronounced. Consequently, the regressions show very high p-values (see S8 Fig), that do not allow the rejection of the null hypothesis. We also found that as the system transitions into a multiple mutations regime, the p-values start to decrease (see S8 Fig). This means that the statistical signal of ERD becomes stronger with more interference. For more than three loci, population sizes of *N* = 10^5^ and *d* ≥ 100 nt, large fractions of the ERD values are detected at statistically significant levels.

These results suggest that under experimental sampling procedures, ERD could be stabilized by interference for systems with more loci and higher *N*. The results also allow to draw some conclusions in the light of the significant associations found in [26], where the p-values of the ERD regressions for the three patients were *p* = 0.0013 (CH40), *p* = 0.00001 (CH77), and *p* = 0.002 (CH58). In our simulations, the fraction of p-values below significance levels increases with higher census population sizes in all the cases considered. Thus, these findings further suggest that if interference is indeed responsible for the observed ERD in HIV patients (and the other assumptions in our simulations hold), observing such low patient p-values is likely at census population sizes of *N* = 10^5^, and unlikely at lower sizes such as *N* = 10^2^.

### Testing escape rate decrease for presence of interference

The support for the notion that interference does entail ERD in the multiple mutations regime led us to analyze whether conversely, observed ERD contains information about the presence of interference during early HIV infection. Several mechanisms, such as different times of emergence of CD8^+^ T cell responses [70], or genetic standing variation in escape mutations segregating in the population might importantly affect ERD. Since in past studies the CD8^+^ T response emergence times [75] and strengths [26] were not predictive of the escape features during early infection (∼4 months), we focused on how standing variation might relate to ERD. To address this question, the effects of standing variation in selective advantages need to be disentangled from interference effects in the pattern of ERD. To this end, we derived a function for the pattern of ERD under the assumptions of free recombination and variation in selective advantages (variation in *s*, or equivalently, *ϵ* of mutations). More specifically, the function predicts what escape rate *ϵ* should be expected at a specific escape time *τ*_50_ under these assumptions.

To obtain this function, we again employed a simple model of early infection. We followed the fate of a single escape mutation through two phases: A neutral phase of viral expansion, free of selective pressures [52] and a second phase with strong selection. In the neutral phase, the escape mutation is subject to expansion dynamics. We calculated the mutation’s frequency at the time of the onset of selection *p*_*s*_. For the selection phase we used the insight that given an *ϵ* of an escape mutation (corresponding to some selective advantage *s*), the frequency trajectory of that escape mutation is fully specified by *p*_*s*_ when going to fixation in the absence of interfering mutations. More specifically, the trajectory follows a logistic curve Eq (1) with escape rate *ϵ* and *f*_0_ = *p*_*s*_. Considering a range of *ϵ* of an escape mutation, will thus account for standing variation of mutations with different *ϵ*.

#### Neutral phase

In the neutral phase, we assumed that mutations accumulate randomly across the genome [50, 52]. Then the probability of finding *n* mutations anywhere in a section of the genome, as derived in [52], is:

$$P(\text{mutations}=n|\text{generations}=g)=\left(\begin{array}{c} g\Lambda \\ n \end{array}\right)\mu^{n}{\left(1-\mu \right)}^{g\Lambda -n},$$ (4)

where *g* is the number of generations, Λ is the length of the genome section in base pairs, and *μ* is the mutation rate per generation per nucleotide.

We can now calculate the expected frequency of an escape mutation in the population at the onset of selection, *p*_*s*_. We assume an epitope to be about ten codons long, i. e. Λ_*s*_ = 30 nt, and the point mutation rate *μ* is 2.15 ⋅ 10^−5^ *nt* ^−1^ [31]. Furthermore, we assume that non-synonymous mutations only appear in 78% of cases, which gives an effective epitope length of Λ_*eff*_ ≡ Λ_*s*_ × 0.78. In HIV we assume a generation time *t*_*g*_ of two days [30, 55–57], such that the number of generations becomes *g* = *t*_*s*_/*t*_*g*_, where *t*_*s*_ is the time in days of the onset of selection post infection.

With these inputs, any epitope will have a mutation at the onset of selection with a probability of

$$p_{s}\left(g,\Lambda_{\text{eff}},\mu \right)≐P\left(\text{mutations}=1|\text{generations}=g\right)=g\Lambda_{\text{eff}}\mu {\left(1-\mu \right)}^{g\Lambda_{\text{eff}}-1},$$ (5)

which results in a value of about *p*_*s*_(*g*, Λ_*eff*_, *μ*) ≈ 1%, assuming that selection starts to operate at *t*_*s*_ ≈ 28 days (i. e. *g* = 14). Note that this value is independent of the census population size *N*. Here, we have assumed that each non-synonymous mutation in an epitope is equivalent to an escape mutation.

#### Selection phase

With a population size of about *N* ≈ 10^4^, a reasonable number for early HIV infection, the number of expected copies of escape mutations at the onset of selection is of the order of one hundred. This number yields valuable information on how to handle the selection phase when interpreted as an establishment size. The population genetics concept of establishment size is mathematically specified: It refers to the lowest population size at which a mutation with selective advantage *s* is assumed to go deterministically to fixation, and is given by 1/*s*. 100 copies is about the establishment size for mutations with *s* ≈ 0.01, which lies within the range of the selective advantages of interest in early HIV infection. Thus, to cover the full range of *s*, we have to assume that some mutations will directly go to fixation at the onset of selection, but others will be lost or not yet be present and go to fixation later.

With this information, we can now fully address the second phase with selection. If the escape mutation considered is destined to go to fixation, it will follow a logistic trajectory to fixation in the absence of interference by other mutations [24, 26, 49, 68]. This trajectory will be determined by the average escape rate *ϵ*, which depends on the selective advantage conferred by the beneficial mutation, and the initial frequency of the mutation *p*_*s*_ at the onset of selection. The time for the frequency of the mutation to reach 50%, the escape time *τ*_50_, will then be the sum of the duration of the neutral phase, *τ*_*n*_, and the time for a mutation to reach 50% starting from *p*_*s*_, Δ*τ*, i. e. *τ*_50_ = *τ*_*n*_ + Δ*τ*.

With Eq (2), Δ*τ* for mutations destined to go to fixation, Δ*τ*_*fix*_, is obtained by setting *f*_0_ = *p*_*s*_:

$$\Delta \tau_{\text{fix}}=\frac{1}{\epsilon }\ln\left(\frac{1-p_{s}}{p_{s}}\right).$$ (6)

To account for vanishing or missing mutations at the onset of selection, we assume that the mutation will reappear or emerge and establish after some delay. To quantify this delay, we use the notion of the *establishment time* *τ*_*e*_, the average time for a mutation with selective advantage *s* to reach establishment size in a population of size *N* with a beneficial mutation rate *μ*_*b*_. It is given by *τ*_*e*_ = 1/(*Nsμ*_*b*_). Again, we set *μ*_*b*_ = 10^−4^. After establishment, the time required for the mutation to reach 50% frequency is given by:

$$\Delta \tau_{\text{est}}=\frac{1}{\epsilon }\ln\left(\frac{1-p_{e}}{p_{e}}\right),$$ (7)

where $p_{e}=\frac{1}{sN}$ is the establishment frequency—the frequency at which the mutation is considered established. With this, the time to reach 50% frequency post infection for a mutation emerging after the onset of selection *τ*_50_ = *τ*_*n*_ + *τ*_*e*_ + Δ*τ*_*est*_.

#### Total time to 50 percent frequency

The time to 50% in either case of direct fixation or reemergence and subsequent fixation should be weighted appropriately. The probability of an escape mutation with frequency *p* and with selective advantage *s* to go to fixation, is given by Kimura’s formula for haploid populations [76]:

$$\Pi \left(p\right)=\frac{1-e^{-2Nsp}}{1-e^{-2Ns}}.$$ (8)

Thus, the case where the mutation directly goes to fixation should be weighted by Π(*p*_*s*_), whereas in the case where the mutation has to reemerge is weighted by 1−Π(*p*_*s*_).

With this, we obtain an expression for expected time to 50% for an escape mutation in the absence of interference:

$$\tau_{50}=\tau_{n}+\Pi \left(p_{s}\right)\Delta \tau_{\text{fix}}+\left(1-\Pi \left(p_{s}\right)\right)\left(\tau_{e}+\Delta \tau_{\text{est}}\right).$$ (9)

Note that in this formula, the third term with factor (1−Π(*p*_*s*_)) will be almost zero if the census population size *N* is large, since then Π(*p*_*s*_) will be close to one.

In expression (9), *τ*_50_ is a function of the escape rate *ϵ* and the selection coefficient *s*, which are similar quantities. To be able to compare the function with data, we require an expression that depends only on *ϵ*, and thus a relation *s*(*ϵ*). Such a relation has been derived by Da Silva [46], based on the dynamics analyzed by Asquith et al. [24]. In this approach, viral strains that do not carry an escape mutation are cleared by CD8^+^ T cells at a fixed rate *k*. Assuming that escape mutations entail a growth reduction rate due to fitness cost *ψ*, the relative fitness of strains targeted by CD8^+^ T cells relative to untargeted is then (1−*ψ*)/(1−*k*), which is equal to *w* in a standard population genetics notation. With *w* = *e* ^*s*^, this yields *s* = ln [(1−*ψ*)/(1−*k*)]. Here, we assume that $\psi \approx 0\;{\text{day}}^{-1}$, since it is usually much smaller than average values of *k* during early infection [24]. We use the assumption that *ϵ* ≈ *k* and therefore $s\approx \ln(\frac{1}{1-\epsilon })$.

With this, Eq (9) becomes:

$$\begin{array}{lll} \tau_{50}\left(\epsilon ,N\right) & = & \tau_{n}+\left(\frac{1-e^{-2Np_{s}\text{ ln}\left(\frac{1}{1-\epsilon }\right)}}{1-e^{-2N\text{ ln}\left(\frac{1}{1-\epsilon }\right)}}\right)\left(\frac{1}{\epsilon }\text{ ln}\left(\frac{1-p_{s}}{p_{s}}\right)\right)\\ & & +\left(1-\frac{1-e^{-2Np_{s}\text{ ln}\left(\frac{1}{1-\epsilon }\right)}}{1-e^{-2N\text{ ln}\left(\frac{1}{1-\epsilon }\right)}}\right)\left(\frac{1}{N\mu_{b}\text{ ln}\left(\frac{1}{1-\epsilon }\right)}+\frac{1}{\epsilon }\text{ ln}\left(N\text{ ln}\left(\frac{1}{1-\epsilon }\right)-1\right)\right) \end{array}$$ (10)

Here, *τ*_50_(*ϵ*, *N*) is well defined for $N\ln(\frac{1}{1-\epsilon })>1$ and *ϵ* < 1. The expression shows the dominant role of the factor 1/*ϵ* in the behavior of *τ*_50_. The inverse function *ϵ*(*τ*_50_,*N*) cannot be obtained analytically, but can be found numerically by optimisation algorithms (we used the R function *optim* [77]).

In Fig 4A, we compare the prediction *ϵ*(*τ*_50_,*N*) from Eq (10) for *N* = 10^4^ and a neutral phase *τ*_*n*_ of 28 days (black line) with data from [20, 26]. The curve does not change significantly for values of *N* larger than 10^4^. The agreement with the data is fair. The curve for *N* = 10^3^ (grey line) is in less good agreement.

Thus, the assumption that escape mutations go to fixation independently of one another is not evidently falsified by comparison with data. To investigate whether this precludes the presence of interference between escape mutations, we compared simulation outcomes from the Wright-Fisher model with data. Fig 4B shows escape rates inferred from similar sample points to those used in [20] for twenty simulation runs of the model. Again, the agreement with the data for the curve with *N* = 10^4^ is fair.

In conclusion, these comparisons do not show an unambiguous sign of interference in the data. Surprisingly, interference effects in the multiple mutations regime seem to shift escape rate estimates along the curve predicted by Eq (10). Hence, linkage information is necessary to clarify the role of interference in the dynamics of early HIV infection.

## Discussion

In this study, we investigated whether multiple interfering concurrent mutations of equal strength can generate patterns of ERD and conversely, whether the presence of ERD in data is indicative of interference effects. To do this, we utilized a computational model of HIV based on a multi-locus Wright-Fisher process with selection, mutation and recombination. The model implements much biological detail in recombination processes as well as in the neutral phase preceding the onset of immune responses.

We found that when utilizing sampling procedures commonly employed in experimental settings, interference causes ERD in multi-epitope systems. However, the presence of ERD is not indicative of interference effects, and could be caused by alternative mechanisms.

One caveat of this study might involve its reliance on the abundance of escapes to study CD8^+^ T cell mediated pressures. A recent study by Roberts et al. following 125 HIV-infected adults for three years, found that a third of patients did not exhibit any escapes, suggesting that escapes might occur less frequently than previously thought [78].

Our study focused on effects produced by beneficial mutations of the same fitness advantage; thus we neglected clonal interference interactions in the specific sense of [33]. According to this narrow notion of *clonal interference*, slightly beneficial mutations are outcompeted by more advantageous mutations that appeared in another background before going to fixation. Crucially, the possible acquisition of an additional rescuing beneficial mutation before extinction is neglected. In contrast, a system generating interference effects under the conditions simulated in this paper is said to evolve in the *multiple mutations regime* [33]. The combination of both of these processes is encompassed in the *concurrent mutations regime* [33, 79].

Whether quasi-asexual systems evolve predominantly by multiple mutations or clonal interference depends critically on the shape of the density distribution of fitness effects [1, 33, 79–81]. Density distributions, which go to zero very slowly for larger positive values of *s* (as 1/*s* ^3^), should show clonal interference effects that resemble sequential fixations [33]. Fitness distributions with a long positive tail, i. e., which are broader than exponential, are expected to display clonal interference [82, 83]. Short-tailed fitness distributions lead to multiple mutations, and the borderline case of an exponential fitness decay combines characteristics of both [80, 81]. Theoretical work suggests that the fitness distribution should be exponential [84–88].

Since the distribution of fitness effects in HIV is not known, our analysis may have ignored important effects of clonal interference. The distribution of beneficial point mutations seems to depend on the selective environment [89], and the fraction of mutations that are beneficial varies among studies [89–91]. However, Desai and Fisher note that systems that satisfy the conditions for the emergence of clonal interference also satisfy the conditions for multiple mutations [33]. This is supported by theoretical results showing that even broad fitness distributions of beneficial mutations can result in a narrow fitness distribution of mutations ultimately going to fixation, averaging out most of the variance in fitness effects [92, 93].

The assessment of the effects of the fitness distribution of mutations in HIV is further complicated by different abundances of distinct epitope-specific CD8^+^ T cell clones. On the one hand, this immunodominance hierarchy could effectively generate fitness distributions with long tails: More abundant CD8^+^ T cell clones could result in stronger selective pressures, and thus higher selective advantages for mutations evading them [94]. The relative CD8^+^ T cell clone abundances would then reflect the differences in selective advantages of each escape mutation. This seems to be suggested by the correlation found in Henn et al. between the escape rate and CD8^+^ T cell response frequencies [22], albeit over long durations of HIV infection of about 1600 days. On the other hand, whether the killing efficacy of CD8^+^ T cells is linearly dependent on their abundance or density is questionable. Models of CD8^+^ T cell action in the spleen show that killing efficacies saturate as a consequence of the mutual impairment of cell recognition and clearance induced by the presence of other CD8^+^ T cells [95, 96]. This saturation effect could level the selective advantages of individual escape mutations, making them very similar. In fact, no correlation between average CD8^+^ T cell abundances and escape rates was found in three other patients [20, 26].

In light of these considerations, this study only elucidates the minimal expectation of interference effects in the concurrent mutations regime, namely the emergence of multiple mutations. The inclusion of clonal interference effects should exacerbate the bias induced by logistic model fitting.

In this study, we ignored fitness costs of escape mutations for several reasons. First, by proposing a very simple model of early HIV dynamics we were able to more clearly identify the sources of some of the effects observed, such as ERD. Second, there seem to exist few high-cost escape mutations [97] and the selective pressures during early HIV are high compared to the fitness costs [24]. This suggests a small role for fitness costs during the phase of HIV infection studied here. Third, compensatory mutations might also be neglected if they go to fixation faster than the escape mutations.

Another caveat stems from CD8^+^ T responses decreasing in strength over time, as suggested by [98]. This behavior could produce the pattern of ERD if all CD8^+^ T cell responses were decreasing simultaneously. However, interference and decreasing CD8^+^ T responses are not mutually exclusive. Hence, a substantial fraction of the ERD pattern could still be produced by interference effects, despite declining immune responses.

In a recent, paper, van Deutekom et al. have offered an alternative explanation for ERD employing an ODE-based virus dynamics model of HIV infection [70]. A mathematical analysis yields that the contribution to killing of each individual CD8^+^ T cell clone must be inversely associated with the breadth of the response. Thus, a broadening response will result in decreasing escape mutation advantages, which should in turn lead to ERD. In our study, we have neglected this effect of immune response broadening. However, interference and CD8^+^ T cell-broadening induced ERD are not mutually exclusive.

Also, unlike van Deutekom et al. we have neglected the early fluctuations of the viral load. This simplification was motivated by the results of our previous work [27], where such fluctuations had little impact on the emergence of interference.

We simulated population sizes up to 10^5^, whereas the number of infected target cells lies between 10^7^ and 10^8^ [28, 29, 69]. Increasing the population size reduces the effects of linkage between beneficial mutations [1, 99]. However, the virus does not necessarily replicate in all of the infected cells, since a large part of the integrated viral DNA could be non-functional [1]. Effective population sizes have been estimated to be lower, namely around 10^5^ [60, 61].

Our results have to be interpreted in the light of the mounting evidence for the role of interference and linked inheritance in a broad range of organisms replicating at large population sizes, ranging from viruses [100], to bacteria [92] to eukaryotes [101]. Our results stress the need for further investigation of the multilocus evolution of HIV during early infection. Accurate assessment of CD8^+^ T cell killing efficacies depends crucially on information about the dynamics of haplotypes constituting the population. If interference effects are important, neglecting linkage will bias estimates of CD8^+^ T cell killing efficacy towards smaller values and underestimate their potential for vaccine development.

That interference effects in early HIV infection could bias the estimation of escape rates has only been recognized in a few publications [27, 34, 49]. ERD has been identified in simulations of stochastic multi-epitope models of HIV integrating an immune response [102]. However, as in [34, 49], interference was not discussed as a possible causal mechanism for the ERD pattern.

In a recent paper, Ganusov et al. presented a mathematical model of HIV within-host evolution which included recombination, but no explicit immune responses [49]. To simulate HIV adaptation following infection of the host, they utilized a Wright-Fisher process based on FFPopSim [48]. The simulations showed that delays increased between the emergence of mutations when compared to fully deterministic dynamics as the effective recombination rate was decreased. The bias in escape rate estimation was particularly pronounced at low sample sizes of around 20.

In a similar paper, Kessinger et al. attempted to estimate escape rates from [20] with a multi-locus model partially allowing for interference effects [34]. The estimation was performed by modeling only a reduced set of haplotypes, assumed to go to fixation sequentially. In line with the predictions under interference, the corrected selective advantages of beneficial mutations were much higher than previously estimated, and showed no evident decrease over time.

Evidence for the coexistence of multiple mutations is emerging especially within single epitopes—called *epitope shattering*. Mutational pathways found in epitope shattering were studied by Leviyang [103] in data from [104]. This shattering phenomenon suggests that if recombination rates are low, interference effects should also appear in inter-epitope mutations. Failure to identify such effects between epitopes would be highly indicative of either a long tailed fitness distribution of escape mutation advantages due to immunodominance or larger recombination rates than previously expected.

Irrespective of the outcome of further research into the matter, the current practice of treating several mutations within an epitope as identical is problematic. The frequencies of these mutations are commonly added together to form the frequency of one overall escape mutation at that same epitope [20, 22, 26]. Interference effects within epitopes could spill over to between-epitope interactions, thereby producing considerable estimation biases, as can be seen when analyzing the data from [22, 43].

## Supporting Information

S1 Text Supporting Information Text detailing the computational methods used for simulations, as well as results for ERD values obtained from different sampling schemes. (PDF)

S1 Fig Schematic representation of the implementation of the recombination procedure in the simulation model. The figure shows an example of production of a recombinant offspring *R* from two parents *A* and *B*. Without loss of generality, *A* is assumed to be the reference sequence, and all mutations or differences are assumed to lie on sequence *B*. As reverse transcriptase proceeds to generate the viral sequence for integration, it will jump to the other sequence with a fixed probability per base pair. The recombinant sequence *R* can be represented by a binary string, which characterizes the information in *R* with respect to reference sequence *A*.
(TIF)

S2 Fig Test of the recombination process in the model utilized for the simulations. The outcome of a simulation with two initial starting haplotypes, (0,0,0) and (1,1,1) at frequencies of 50% is shown. The population size is *N* = 5 × 10^5^, and mutation effects are not present. In the haplotype dynamics all of the haplotypes are generated by recombination, and equilibrate at linkage equilibrium with equi-partitioned frequencies.
(TIF)

S3 Fig Two examples of the escape dynamics in the Wright-Fisher model with selection, showing how the logistic model fits and the calculation of ERD were carried out. The upper row shows the time courses of epitope frequencies for two examples (A and B), of simulation runs of the Wright-Fisher model with selection and recombination. The simulations included six loci, with *N* = 10^4^, *μ*_*b*_ = 10^−4^ per locus per generation, *s* = 0.5, no neutral phase and an inter-locus distance of 3000 nt. In each simulation run, a pair of values (*ϵ*_*l*_,*τ*_50, *l*_) is estimated from the logistic fit to each epitope *l* going to fixation. A regression *log*_10_(*ϵ*) = *a* + *b* ⋅ *τ*_50_ is performed on the value pairs extracted from the fits in A and B (C and D, respectively). The p-values correspond to the test of the null hypothesis *H*_0_: *b* = 0.
(TIF)

S4 Fig Median of the per-simulation-run residual sum of squares median of logistic fits to escape mutation frequencies in the Wright-Fisher simulations with selection. Within each simulation, we fitted a logistic type curve Eq (1) to the escape mutation frequencies of each epitope. The median residual sum of squares of these logistic fits was taken across these escapes, i. e., one median residual sum of squares value per simulation. The median of these values (inferred from 100 simulations) is shown for *L* = 3, 4, 5, 6 loci in rows A-C, D-F, G-I and J-L, respectively. The values for inter-mutation distances of *d* = 0 (complete linkage), *d* = 100 and *d* = 3000 nt are shown in columns A-J, B-K, C-L, respectively. The red line (green, blue lines) correspond to neutral phases of 0 (20, 28) days, respectively.
(TIF)

S5 Fig ERD values inferred with sampling frequency of 30 days versus population sizes, different neutral phase durations, linkage strengths and numbers of loci. Rows: Simulations were performed for *L* = 3, 4, 5, 6 loci shown in rows A-C, D-F, G-I and J-L, respectively. Columns: In each row the effect of loosening linkage on ERD is shown for inter-mutation distances of *d* = 0 (complete linkage), *d* = 100 and *d* = 3000 nt. Colors: The thick red line (green, blue lines) and the thin light-red (light-green, light-blue) lines show the median and 25 and 75 percentiles of ERD inferred from 100 simulations with neutral phase of 0 (20, 28) days, respectively. Selective coefficients are *s* = 0.5 for all beneficial mutations, and the epitope mutation rate is *μ*_*b*_ = 10^−4^ per locus per generation. 30 samples were taken at fixed time periods starting from the onset of selection until 400 days (roughly every 13 days).
(TIF)

S6 Fig Correlation between ERD value estimates obtained by sampling at times as in empirical studies and fixed sampling periods (roughly every 13 days). Rows A-C) show inter-mutation distances of 0 nt, D-F) 100 nt and G-I) 3000 nt, respectively. Columns show simulations run with neutral periods of 0 days, 20 days and 28 days before elicitation of selection pressures. Population sizes were set to *N* = 10^5^. The fitted lines result from a Theil-Sen estimator (red) and a linear regression (green). Their slopes are given in the insets with corresponding confidence intervals.
(TIF)

S7 Fig Density distribution of median escape times estimated from simulations with distinct numbers of loci, neutral phase durations and recombination rates. Rows show the effect of increasing number of loci *L* = 3, 4, 5, 6, respectively. Columns show the effect of loosening linkage for inter-mutation distances of *d* = 0 (complete linkage), *d* = 100 and *d* = 3000 nt. Colors: The red line (green, blue lines) shows the density distribution of median escape times inferred from simulations with neutral phase of 0 (20, 28) days, respectively. Median escape times were inferred from simulations (see Materials and Methods) for experiment-like sample times in 100 individual repeats. The vertical lines denote the median of the respective distributions. Selection coefficients were set to *s* = 0.5 for all beneficial mutations, and the epitope mutation rate was set to *μ*_*b*_ = 10^−4^ per locus per generation.
(TIF)

S8 Fig P-values for the null-hypothesis of zero ERD value versus population sizes for different numbers of loci, neutral phase lengths and linkage strengths. Rows: The ERD values were inferred from escapes for *L* = 3, 4, 5, 6 loci, shown in rows A-C, D-F, G-I and J-L, respectively. Columns: The effect of loosening linkage on ERD is shown for inter-mutation distances of *d* = 0 (complete linkage), *d* = 100 and *d* = 3000 nt. Colors: The red line (green, blue lines) and the lines of light-red (light-green, light-blue) color show the median and 25 and 75 percentiles of the p-value of 100 simulations with neutral phase of 0 (20, 28) days, respectively. In the simulations, each beneficial mutation conferred a selective advantage of *s* = 0.5. The beneficial mutation rate is *μ*_*b*_ = 10^−4^ per locus per generation. Samples were taken roughly as described in [20].
(TIF)
