# Supplementary material for: Investigating the Consequences of Interference between Multiple CD8+ T Cell Escape Mutations in Early HIV Infection
Source: PLoS Comput Biol. 2016 Feb 1;12(2):e1004721. doi: 10.1371/journal.pcbi.1004721 (PMC4735108; doi:10.1371/journal.pcbi.1004721)
Supplement: S1 Text — (PDF) [file pcbi.1004721.s001.pdf]

Supporting Information

Investigating the consequences of interference between multiple $CD8^+$ T cell escape mutations in early HIV infection

Victor Garcia[1,*], Marcus W. Feldman[2], and Roland R. Regoes[1]

[1]Institute of Integrative Biology, ETH Zurich, Universitätstr. 16, CH-8092 Zurich, Switzerland

[2]Department of Biological Sciences, Stanford University, Stanford, California, USA

[*]*corresponding author:* P: +41-44-633 60 34, email: victor.garcia_palencia@alumni.ethz.ch

Effects of different sampling sizes on escape rate decrease

To investigate the effects of sampling schemes on escape rate decrease, we performed the same inference procedures for escape rates as explained in the main manuscript, but with equally spaced sample times. Figure S5 Fig shows the ERD values inferred from different numbers of loci (rows), different neutral phase durations (colors in parts of figures) and linkage strengths (columns). The patterns in the figure are very similar to those shown for experimental sampling times in Figure 2 of the manuscript. As the system transitions to a larger population size, interference effects become important, and ERD values become negative. The transition between the two regimes is more clearly visible with equally-spaced than experimental sampling times. We also observe that more loci reduce the impact of interference on escape rate decrease. The effect of the neutral phase preceding the onset of selection is almost negligible, indicating a small role of soft sweeps due to standing variation.

Mutation

In the implementation of the model, following each generation step with selection a procedure is applied to add mutations to the population. This procedure acts on the offspring population of each individual strain or haplotype, $\mathbf{i}$. We assume that mutations occur at a rate $\mu = 2.15 \cdot 10^{-5}$ per replication per base pair [1].

The assumption of a constant mutation rate across the genome entails that the probability that an offspring contains k mutations is given by a binomial distribution:

$$p_k = \binom{L}{k} \mu^k (1-\mu)^{L-k}, \tag{1}$$

where L denotes the sequence length simulated, or equivalently, the number of loci in the sequence.

With this information, we can calculate the proportions of single mutants, double mutants and so forth stemming from one particular ancestral strain in the previous generation. Since this probability distribution applies for each offspring stemming from a particular ancestral haplotype, the number of its offspring-strains containing k mutations can be obtained easily. This is achieved by sampling with replacement with probabilities p_k from the offspring population of an individual sequence type. Sampling with replacement was implemented as a multinomial distribution with event probabilities p_k as implemented in the Math.Net Numerics library.

Subsequently, a loop over the subpopulation of offspring with k mutations is carried out. For the sub-population with $k = 1$ mutations, which is likely to constitute the largest number of mutants, we apply further procedures. We sample from that population with a multinomial distribution with equal event probabilities $p_{l,\text{single}} = 1/L$, with $l \in \{1, \dots, L\}$. We thereby obtain a vector of length L of single-mutant sequences carrying mutations at loci l. This procedure can therefore generate all of the offspring sequences differing from the ancestor by a single mutation. The newly formed haplotypes or strains are then reintegrated into the population for the next generation step to be carried out.

The sub-populations characterized by $k > 1$ mutations in their sequences are handled differently. Each of the multiple-mutation strains is dealt with individually. For each strain the positions of the mutations in the genome are randomly sampled (again, the assumption is that all of the loci are equally susceptible to mutation). When a locus is chosen for mutation, the zero entry is replaced by a one.

Implementation of recombination

In the model here presented, recombination operates in a population containing different strains. Naïvely, we could think of each strain as occupying one target cell. The number of cells infected with particular strains is tracked by the simulation model over the time of the simulation.

However, in HIV/SIV, recombination has been shown to arise from double infections of single cells. We therefore assume that a certain proportion of cells has been infected with two haplotypes. These two strains both inserted their genetic information into the doubly infected cell's nucleus, which then starts to produce the offspring RNA. This RNA is subsequently released into the the cytoplasm. There, two different strains may be packaged into one single viral protein envelope, which is then discharged into the host as a new virion. Recombination is only viable if a virion packaging two different strains

infects a new host cell. Recombination effectively occurs after the double-packaged virion capsule has entered a new cell, and reverse transcriptase occurs within it. In our model, we assume that all of the processes leading to recombination occur in one generation.

Since our model is designed to track cells infected with one single haplotype only, i.e., a population of infected cells, recombination was set up to act only upon a fraction of the population, assumed to represent the population of doubly infected cells. That fraction is determined by the proportion of doubly infected cells in a counter-intuitive way, and sampled from the population of infected cells without replacement.

To simulate recombination in our model, we assume that a proportion p_{model} of all strains of our modeled population is sampled to belong to a population of doubly infected cells. In contrast, infected cells in a real biological setting within the host are assumed to permanently sustain a proportion p of cells that are doubly infected. These two proportions p_{model} and p are different.

To clarify this point, we distinguish between the total number of infected cells in the host denoted by n_{eff}, and the total number of cells tracked in our model n_{model}. Since the population list in the model only considers single-infected cells, but a fraction p of the entire population of infected cells is infected with *two* strains in reality, there must exist a mismatch between the two. The conclusion is that there has to exist a proportion of the modeled population p_{model} that resides in doubly infected cells. In other words, since the real number of infected cells within a host n_{eff} contains cells with two instead of one haplotype infecting them, that number ought to be smaller than the one specified in the model, n_{model}.

The proportion p_{model} is also the fraction of cells in which recombination may occur. We derive a relation for p_{model} that depends on the proportion of doubly infected cells p in an effective population n_{eff} of host cells. To do this, we first note that the number of cells that are doubly infected with two strains in a real host is given by $p \cdot n_{\text{eff}}$. The relation between the number of infected cells in the model n_{model} and the total number of infected cells n_{eff} is given by:

$$n_{\text{model}} = (n_{\text{eff}} - p \cdot n_{\text{eff}}) + 2 \cdot p \cdot n_{\text{eff}} = (1 + p)n_{\text{eff}}. \tag{2}$$

Furthermore, the proportion of the singly infected cells that has to be mapped to doubly infected cells p_{model} is given by:

$$p_{\text{model}} = \frac{2pn_{\text{eff}}}{n_{\text{model}}} = \frac{2p}{1 + p}, \tag{3}$$

where the second equality is achieved by virtue of equation (2). The value of the proportion p has been estimated experimentally to be around 1% [2–6].

In the computational model, recombination is therefore only applied to a fraction p_{model} of the population. This sub-population is sampled from the population each generation. We term this sub-population the *recombining sample*, whose sequences recombine randomly with sequences within the same sample.

Since recombination acts on pairs of sequences, it is necessary to identify frequencies of the combinations of pairs of strains which will form and recombine. To do this, a

matrix of to-be-recombined-sequences (pairs) stemming from the recombining sample is constructed. The entire recombination procedure essentially involves five steps, out of which the first three lead to the formation of the pair-matrix. In the following, these five steps are described in more detail.

First, the recombining sample is subdivided into two sub-populations of partners X and Y, where **x** and **y** are types (haplotypes) within X and Y, respectively. The sets X and Y are organized into lists, each providing the candidate partners for a particular recombination event. These subpopulations are generated by sampling half of the population of the recombining sample without replacement, which is X. The rest is Y.

Second, given two sub-populations –containing the recombination candidates for partners **x** and **y**, respectively– the frequencies of general pairs of type (**i**,**j**) for completely random mixing (where **i**,**j** denote the haplotypes contained in the samples X and Y respectively) need to be formed. The subdivision of the original recombination sample into two halves has potentially created unequal distributions of haplotypes within X and Y. Therefore, the probability for the generation of a pair (**i**,**j**) out of X and Y is non-trivial. To resolve this issue, a loop through the haplotypes of sample Y (**j**) is performed. For each **j**, a sample is taken without replacement from X exactly as many times as haplotype **j** occurs in Y. The extracted subsample is then removed from X, and the loop proceeds to the next haplotype in Y. A mixing matrix of pair numbers (**i**,**j**) is generated sequentially by this procedure, creating a number of n(**i**,**j**) of pairs. With this method we fill a mixing matrix containing all recombining pairs to be considered.

Third, the generated mixing matrix is converted into a triangular matrix, since the action of recombination on a pair (**i**,**j**) is equivalent to its action on a pair (**j**,**i**). In this triangular matrix, pair (i,j) has a frequency denoted by n'(**i**,**j**), which means that there exist n'(**i**,**j**) doubly infected cells that contain one strain **i** and one strain **j**.

Fourth, the program loops through all non-equal pairs (**i**,**j**) (where $\mathbf{i} \neq \mathbf{j}$) and produces new recombinant strains (see below for more details). The recombinant strains are then added to a reinsertion sample, which also includes the pairs of equal strains.

Fifth, the reinsertion sample is returned to the original population.

Production of recombinant offspring

The recombinant strains are generated by a separate method which implements the action of reverse transcriptase with as much biological detail as possible. In order to realistically recreate recombination in HIV it is necessary to calculate the probability of generating a recombinant genotype R, out of two recombining strains A and B.

After insertion of a pair of distinct RNA-strands into the cell cytoplasm by a virion, reverse transcriptase begins to produce the viral DNA. We assume reverse transcriptase starts from one end of the strain sequence and proceeds towards the other end. Without loss of generality, we assume that this occurs at the 0-th entry or locus of the simulated sequences. We further assume that each recombinant partner sequence is equally likely to be the sequence where reverse transcriptase is initiated. To account for that, one of the two sequences is randomly chosen at the end of the recombination procedure. For

simplicity, we assume that reverse transcriptase starts at a parent recombinant strain A, and can switch templates to the recombinant parent B and back (see S1 Fig).

We then assume that as reverse transcriptase proceeds along one partner sequence, each base pair has a probability ρ of inducing a template switch of the reverse transcriptase to the other sequence. The number of switches occurring before the transcription reaches a particular position m in one of the two sequences is therefore described by a Binomial Distribution with m trials and success probability ρ. The parameter ρ has been estimated to be 3×10^{-4} per bp per generation [7, 8]. The number of successes k is therefore binomially distributed.

In general, the two parent recombinant sequences differ at a finite number of positions. For simplicity, we picture these differences to be characterized by mutations all contained in sequence B, since they are viewed as mutations with respect to sequence A (see S1 Fig). For a mutation located at a position m_1 in recombinant partner B to be included into the recombinant offspring, the transcriptase process needs to have switched an odd number of times between the sequences before it reaches that position. The probability for an odd number of successes is given by:

$$\Pr(k\,\text{odd}, m) = (1-\rho)^m \cdot \left(\left(\frac{\rho}{1-\rho} + 1 \right)^m - \left(1 - \frac{\rho}{1-\rho} \right)^m \right) = 1 - (1-2\rho)^m. \quad (4)$$

On the other hand, for the even case, we have $\Pr(k\,\text{even}, m) = 1 - \Pr(k\,\text{odd}, m)$.

Recombinant offspring sequences R can then be characterized by whether they contain the mutations that distinguish sequences A and B by use of binary sequences. For example, $R = (0, 1, 0)$ denotes a recombinant offspring which does not contain the first mutation at locus m_1, contains the second at m_2, but does not contain the third at m_3. The probability of generating R depends on whether the mutation sites have been integrated into R by the action of reverse transcriptase, which is in turn dependent on the relative distances of the mutations from each other.

R is therefore going to be generated with a probability of:

$$\Pr(R) = \Pr(k\,\text{even}, m_1 - 0) \cdot \Pr(k\,\text{odd}, m_2 - m_1) \cdot \Pr(k\,\text{odd}, m_3 - m_2). \quad (5)$$

The probability that reverse transcriptase includes the next mutation is dependent on the distance between mutations. We will call the probability that reverse transcriptase switches between two particular sites $p_s(\Delta m)$ from now on, the probability of not doing so will be denoted $p_r(\Delta m)$, where Δm is the distance to the last mutation or the sequence start. Hence, the probability of producing R is a concatenation of these probabilites (always considering the relative distances to other mutations).

To give an example of how the probability to generate a recombinant offspring sequence R is computed, let the offspring R be encoded by (0, 1, 0, 1, 1) (see S1 Fig). First, the sub-probabilites $p_r(\Delta m)$ and $p_s(\Delta m)$ are calculated for all relative mutation distances Δm. Subsequently, a reference encoding Q to R is generated as follows: Q starts with 0 and concatenates the original encoding to the zero leaving the last digit of R out. Hence, Q is given by $(0, 0, 1, 0, 1)$. In order to find the right sequence of p_r's and p_s's we

calculate $R \neq Q$ between the two strains in a bitwise fashion: $(0,1,1,1,0)$. For each 0 a p_r is placed in the product, for each 1 a p_s. The probability for R to be produced is therefore: $p_r(m_1 - 0) \cdot p_s(m_2 - m_1) \cdot p_s(m_3 - m_2) \cdot p_s(m_4 - m_3) \cdot p_r(m_5 - m_4)$.

An important consequence of this approach is that we have only calculated probabilities with respect to one particular sequence A. Equivalently, reverse transcriptase could have been initiated at sequence B. To account for this, it is important to note that the probability for a haplotype to be produced by recombination (with respect to reference sequence A), only differs from the probability to produce the same haplotype starting reverse transcriptase from B by first probability entry $p_{r,s}(m_1 - 0)$. That is, haplotype (0, 1, 0, 1, 1) as defined by the reference sequence A is produced with probability $p_r(m_1 - 0) \cdot p_s(m_2 - m_1) \cdot p_s(m_3 - m_2) \cdot p_s(m_4 - m_3) \cdot p_r(m_5 - m_4)$ if reverse transcriptase starts from the sequence A. The same the haplotype (still with respect to reference sequence A) is produced with probability $p_s(m_1 - 0) \cdot p_s(m_2 - m_1) \cdot p_s(m_3 - m_2) \cdot p_s(m_4 - m_3) \cdot p_r(m_5 - m_4)$ if reverse transcriptase starts at sequence B. Since these two events are mutually exclusive and happen with equal probability 1/2 (reverse transcriptase starts at A with 50% probability or B with 50% probability), the probability for (0, 1, 0, 1, 1) is their sum, which is $\frac{1}{2} \cdot p_s(m_2 - m_1) \cdot p_s(m_3 - m_2) \cdot p_s(m_4 - m_3) \cdot p_r(m_5 - m_4)$.

For simplicity, the computational model utilized in the simulations assumed a constant distance between adjacent loci $d = m_{l+1} - m_l$, for all $l \in \{1, \ldots, L\}$. Simulations were run for $d \in \{0, 10, 100, 3000\}$.

The action of recombination is shown in S2 Fig. The figure shows the evolution of a system with two initial ancestral strains, (0,0,0) and (1,1,1). No mutation or selective forces are considered, just drift and recombination. As expected, all eight possible haplotypes are produced by recombination over time, and the system reaches a state of linkage equilibrium.

Hence, with information about the probability of a recombinant strain R to emerge from a recombination event, the production of the new recombinants is straight-forward. The new recombinants were sampled without replacement (via a multidimensional hypergeometric distribution) from each combination of parental pairs. When sampling, the corresponding probabilities for each potential recombinant offspring were calculated by means of equation (5). The number of possible new recombinants between a pair $(\mathbf{x}, \mathbf{y})$ is simply given by $2^d(\mathbf{x}, \mathbf{y})$, where $d(\mathbf{x}, \mathbf{y})$ is the Hamming distance between $\mathbf{x}$ and $\mathbf{y}$.

References

[1] Mansky LM, Temin HM. Lower in vivo mutation rate of human immunodeficiency virus type 1 than that predicted from the fidelity of purified reverse transcriptase. Journal of Virology. 1995 Aug;69(8):5087–5094.

[2] Jung A, Maier R, Vartanian JP, Bocharov G, Jung V, Fischer U, et al. Recombination: Multiply infected spleen cells in HIV patients. Nature. 2002;418(6894):144–144.

[3] Josefsson L, Palmer S, Casazza J, Ambrozak D, Kearney M, Shao W, et al. Analysis

of HIV DNA molecules in paired peripheral blood and lymph node tissue samples from chronically infected patients. In: Antiviral Therapy. vol. 15. INT MEDICAL PRESS LTD 2-4 IDOL LANE, LONDON EC3R 5DD, ENGLAND; 2010. p. A41–A41.

[4] Neher RA, Leitner T. Recombination rate and selection strength in HIV intra-patient evolution. PLoS Computational Biology. 2010;6(1):e1000660.

[5] Batorsky R, Kearney MF, Palmer SE, Maldarelli F, Rouzine IM, Coffin JM. Estimate of effective recombination rate and average selection coefficient for HIV in chronic infection. Proceedings of the National Academy of Sciences. 2011;108(14):5661–5666.

[6] Mostowy R, Kouyos RD, Fouchet D, Bonhoeffer S. The role of recombination for the coevolutionary dynamics of HIV and the immune response. PloS One. 2011;6(2):e16052.

[7] Jetzt AE, Yu H, Klarmann GJ, Ron Y, Preston BD, Dougherty JP. High rate of recombination throughout the human immunodeficiency virus type 1 genome. Journal of Virology. 2000;74(3):1234–1240.

[8] Zhuang J, Jetzt AE, Sun G, Yu H, Klarmann G, Ron Y, et al. Human immunodeficiency virus type 1 recombination: rate, fidelity, and putative hot spots. Journal of Virology. 2002;76(22):11273–11282.

Supporting Information Legends

S1 Fig

Schematic representation of the implementation of the recombination procedure in the simulation model. The figure shows an example of production of a recombinant offspring R from two parents A and B. Without loss of generality, A is assumed to be the reference sequence, and all mutations or differences are assumed to lie on sequence B. As reverse transcriptase proceeds to generate the viral sequence for integration, it will jump to the other sequence with a fixed probability per base pair. The recombinant sequence R can be represented by a binary string, which characterizes the information in R with respect to reference sequence A.

S2 Fig

Test of the recombination process in the model utilized for the simulations. The outcome of a simulation with two initial starting haplotypes, (0,0,0) and (1,1,1) at frequencies of 50% is shown. The population size is $N = 5 \times 10^5$, and mutation effects are not present. In the haplotype dynamics all of the haplotypes are generated by recombination, and equilibrate at linkage equilibrium with equi-partitioned frequencies.

S5 Fig

ERD values inferred with sampling frequency of 30 days versus population sizes, different neutral phase durations, linkage strengths and numbers of loci. Rows: Simulations were performed for $L = 3, 4, 5, 6$ loci shown in rows A-C, D-F, G-I and J-L, respectively. Columns: In each row the effect of loosening linkage on ERD is shown for inter-mutation distances of $d = 0$ (complete linkage), $d = 100$ and $d = 3000$ nt. Colors: The thick red line (green, blue lines) and the thin light-red (light-green, light-blue) lines show the median and 25 and 75 percentiles of ERD inferred from 100 simulations with neutral phase of 0 (20, 28) days, respectively. Selective coefficients are $s = 0.5$ for all beneficial mutations, and the epitope mutation rate is $\mu_b = 10^{-4}$ per locus per generation. 30 samples were taken at fixed time periods starting from the onset of selection until 400 days (roughly every 13 days).
