# Supplementary figures and images for: Investigating the Consequences of Interference between Multiple CD8+ T Cell Escape Mutations in Early HIV Infection
Source: PLoS Comput Biol. 2016 Feb 1;12(2):e1004721. doi: 10.1371/journal.pcbi.1004721 (PMC4735108; doi:10.1371/journal.pcbi.1004721)

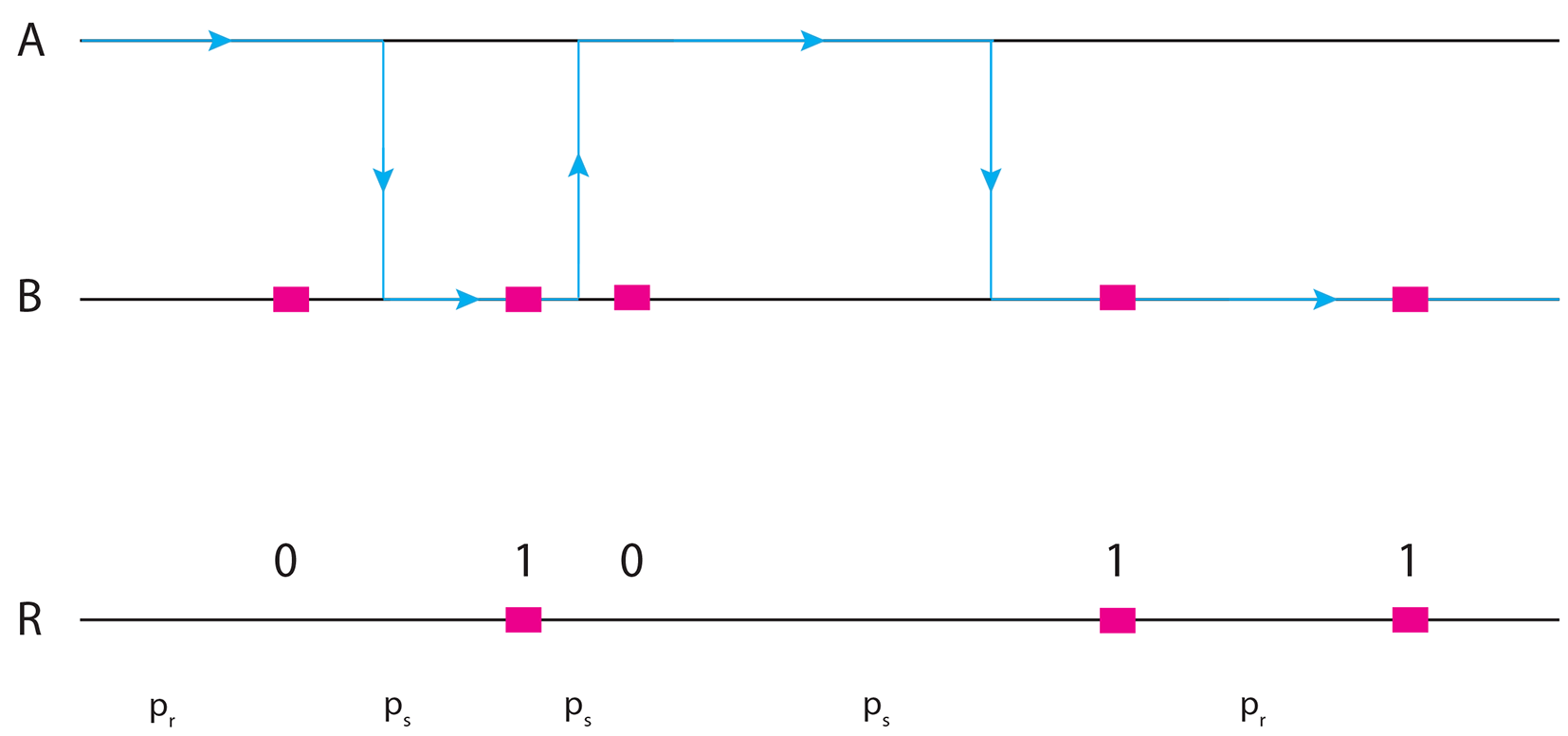

Supplement: S1 Fig — The figure shows an example of production of a recombinant offspring R from two parents A and B. Without loss of generality, A is assumed to be the reference sequence, and all mutations or differences are assumed to lie on sequence B. As reverse transcriptase proceeds to generate the viral sequence for integration, it will jump to the other sequence with a fixed probability per base pair. The recombinant sequence R can be represented by a binary string, which characterizes the information in R with respect to reference sequence A. (TIF) [file pcbi.1004721.s002.tif]

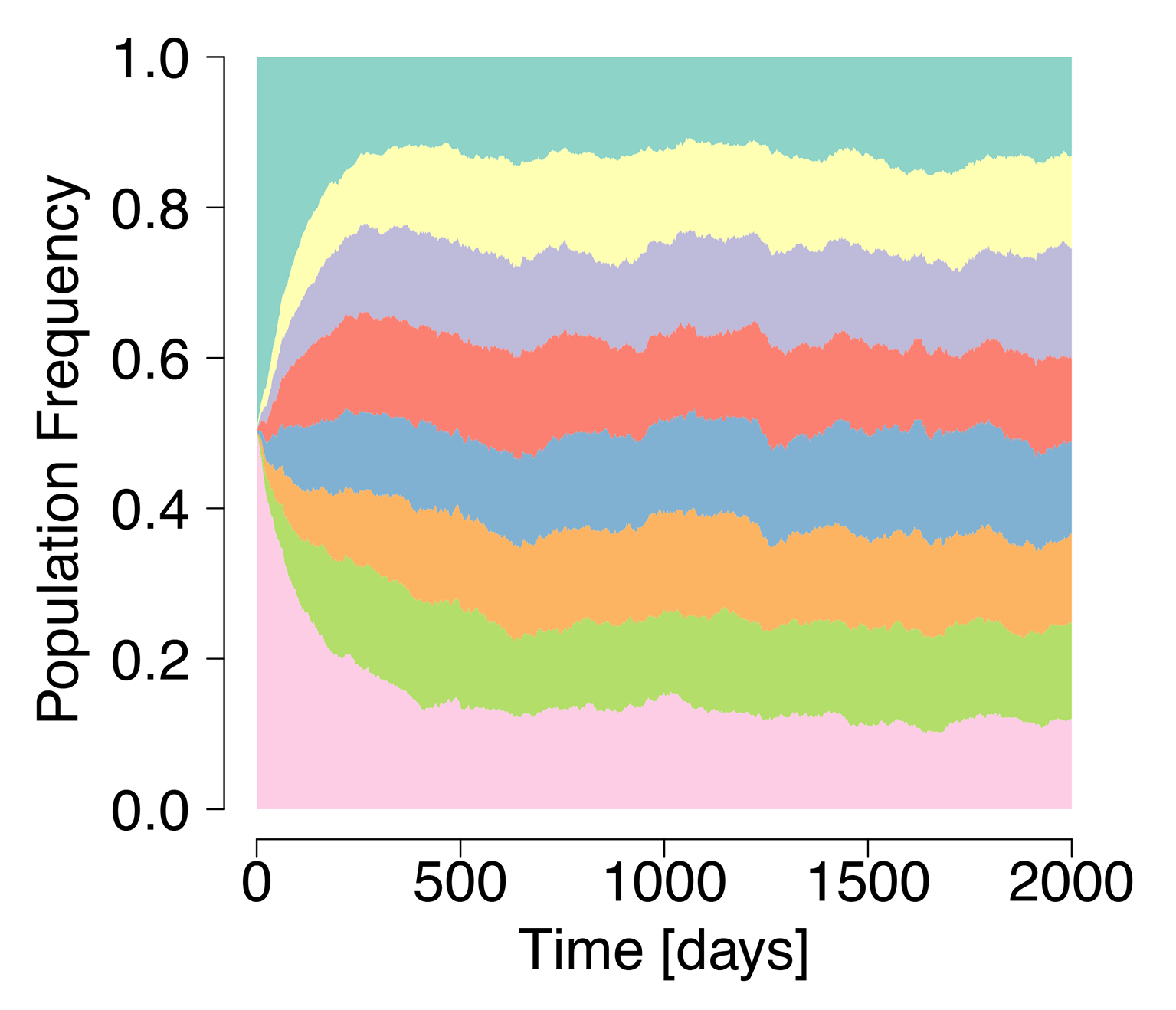

Supplement: S2 Fig — The outcome of a simulation with two initial starting haplotypes, (0,0,0) and (1,1,1) at frequencies of 50% is shown. The population size is N = 5 × 10^5^, and mutation effects are not present. In the haplotype dynamics all of the haplotypes are generated by recombination, and equilibrate at linkage equilibrium with equi-partitioned frequencies. (TIF) [file pcbi.1004721.s003.tif]

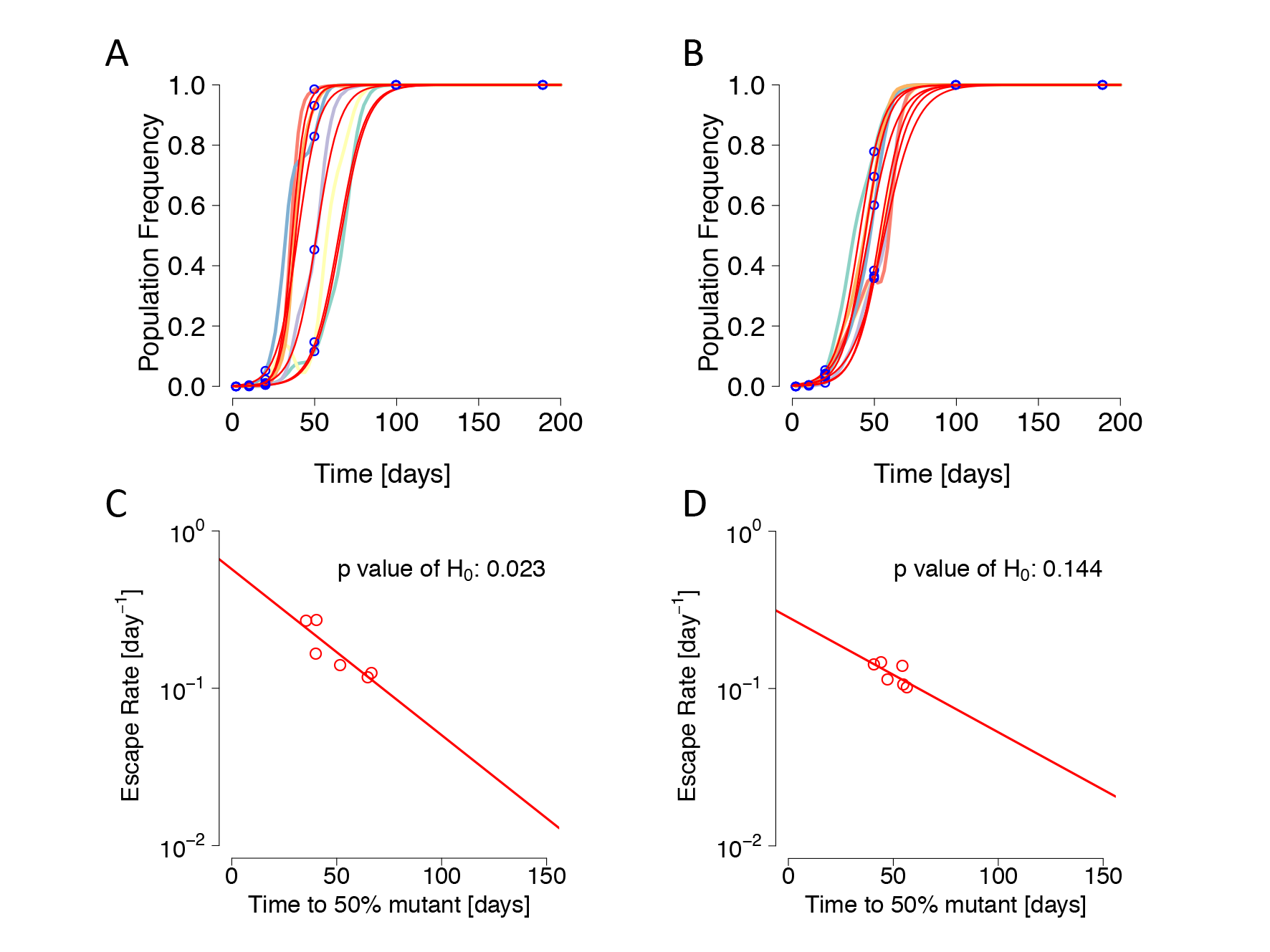

Supplement: S3 Fig — The upper row shows the time courses of epitope frequencies for two examples (A and B), of simulation runs of the Wright-Fisher model with selection and recombination. The simulations included six loci, with N = 104, μ b = 10−4 per locus per generation, s = 0.5, no neutral phase and an inter-locus distance of 3000 nt. In each simulation run, a pair of values (ϵ l,τ 50, l) is estimated from the logistic fit to each epitope l going to fixation. A regression log 10(ϵ) = a + b ⋅ τ 50 is performed on the value pairs extracted from the fits in A and B (C and D, respectively). The p-values correspond to the test of the null hypothesis H 0: b = 0. (TIF) [file pcbi.1004721.s004.tif]

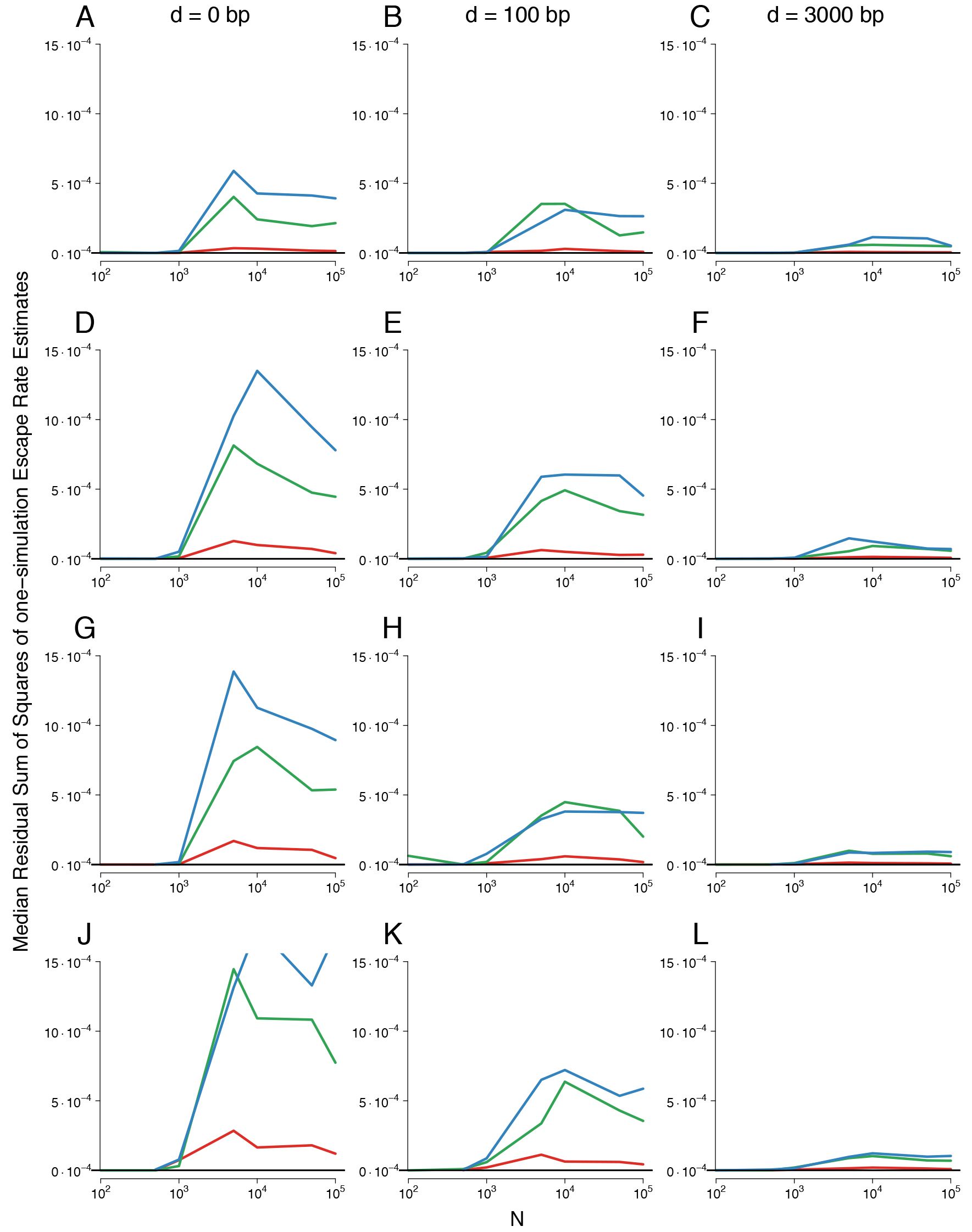

Supplement: S4 Fig — Within each simulation, we fitted a logistic type curve Eq (1) to the escape mutation frequencies of each epitope. The median residual sum of squares of these logistic fits was taken across these escapes, i. e., one median residual sum of squares value per simulation. The median of these values (inferred from 100 simulations) is shown for L = 3, 4, 5, 6 loci in rows A-C, D-F, G-I and J-L, respectively. The values for inter-mutation distances of d = 0 (complete linkage), d = 100 and d = 3000 nt are shown in columns A-J, B-K, C-L, respectively. The red line (green, blue lines) correspond to neutral phases of 0 (20, 28) days, respectively. (TIF) [file pcbi.1004721.s005.tif]

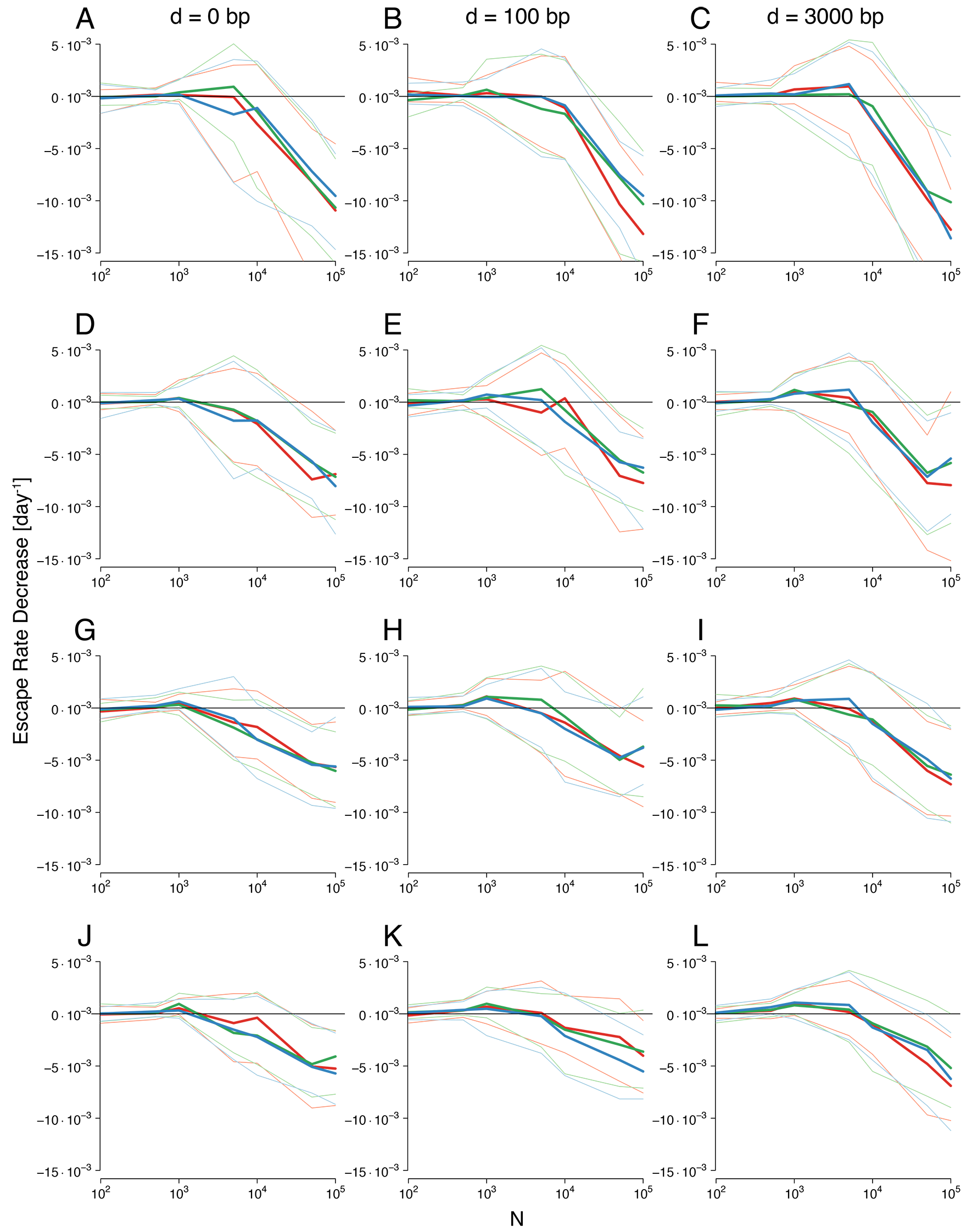

Supplement: S5 Fig — Rows: Simulations were performed for L = 3, 4, 5, 6 loci shown in rows A-C, D-F, G-I and J-L, respectively. Columns: In each row the effect of loosening linkage on ERD is shown for inter-mutation distances of d = 0 (complete linkage), d = 100 and d = 3000 nt. Colors: The thick red line (green, blue lines) and the thin light-red (light-green, light-blue) lines show the median and 25 and 75 percentiles of ERD inferred from 100 simulations with neutral phase of 0 (20, 28) days, respectively. Selective coefficients are s = 0.5 for all beneficial mutations, and the epitope mutation rate is μ b = 10−4 per locus per generation. 30 samples were taken at fixed time periods starting from the onset of selection until 400 days (roughly every 13 days). (TIF) [file pcbi.1004721.s006.tif]

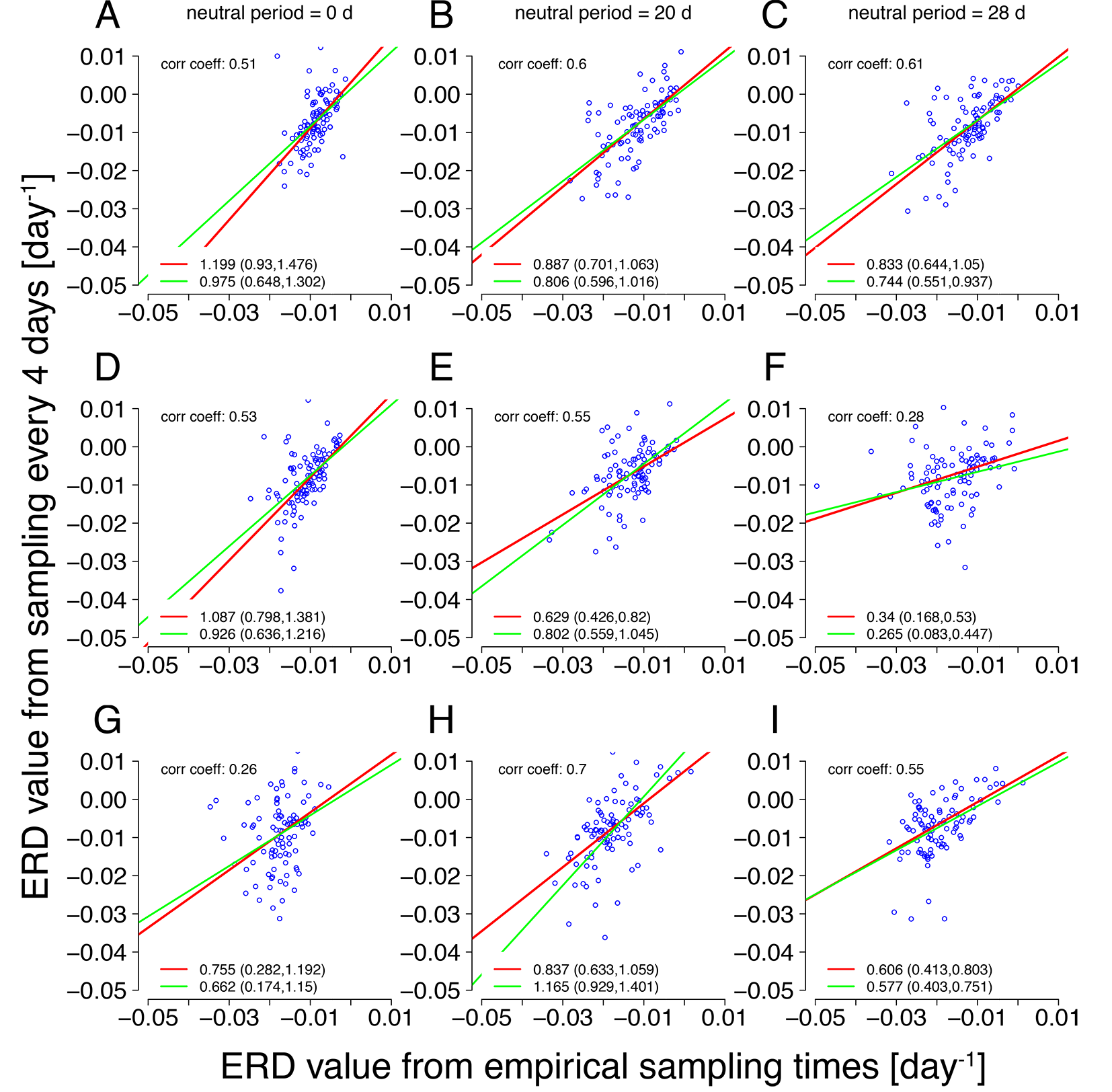

Supplement: S6 Fig — Rows A-C) show inter-mutation distances of 0 nt, D-F) 100 nt and G-I) 3000 nt, respectively. Columns show simulations run with neutral periods of 0 days, 20 days and 28 days before elicitation of selection pressures. Population sizes were set to N = 105. The fitted lines result from a Theil-Sen estimator (red) and a linear regression (green). Their slopes are given in the insets with corresponding confidence intervals. (TIF) [file pcbi.1004721.s007.tif]

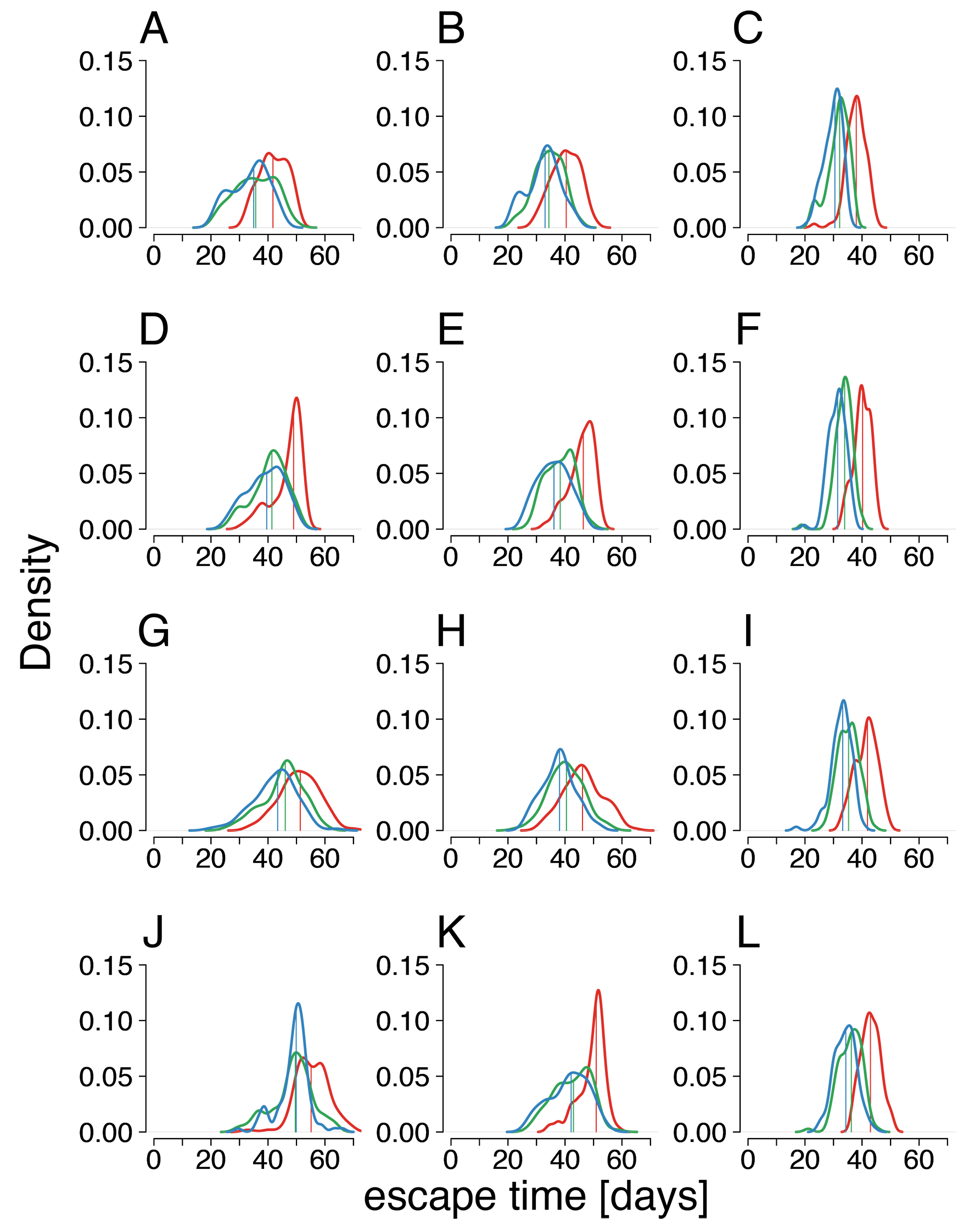

Supplement: S7 Fig — Rows show the effect of increasing number of loci L = 3, 4, 5, 6, respectively. Columns show the effect of loosening linkage for inter-mutation distances of d = 0 (complete linkage), d = 100 and d = 3000 nt. Colors: The red line (green, blue lines) shows the density distribution of median escape times inferred from simulations with neutral phase of 0 (20, 28) days, respectively. Median escape times were inferred from simulations (see Materials and Methods) for experiment-like sample times in 100 individual repeats. The vertical lines denote the median of the respective distributions. Selection coefficients were set to s = 0.5 for all beneficial mutations, and the epitope mutation rate was set to μ b = 10−4 per locus per generation. (TIF) [file pcbi.1004721.s008.tif]

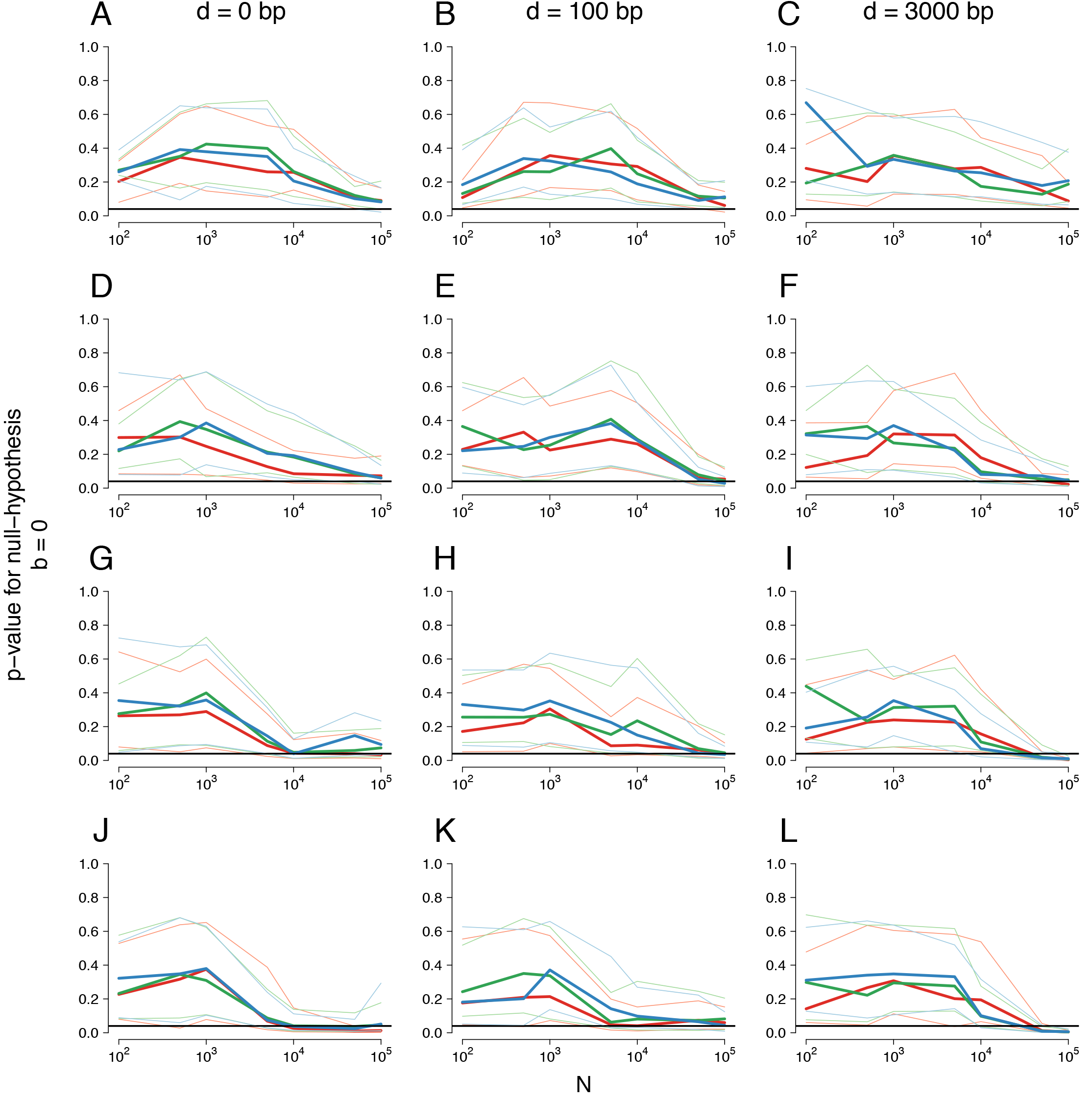

Supplement: S8 Fig — Rows: The ERD values were inferred from escapes for L = 3, 4, 5, 6 loci, shown in rows A-C, D-F, G-I and J-L, respectively. Columns: The effect of loosening linkage on ERD is shown for inter-mutation distances of d = 0 (complete linkage), d = 100 and d = 3000 nt. Colors: The red line (green, blue lines) and the lines of light-red (light-green, light-blue) color show the median and 25 and 75 percentiles of the p-value of 100 simulations with neutral phase of 0 (20, 28) days, respectively. In the simulations, each beneficial mutation conferred a selective advantage of s = 0.5. The beneficial mutation rate is μ b = 10−4 per locus per generation. Samples were taken roughly as described in [20]. (TIF) [file pcbi.1004721.s009.tif]
